# LncTUG1 contributes to the progression of hepatocellular carcinoma via the miR-144-3p/RRAGD axis and mTOR/S6K pathway

**DOI:** 10.1038/s41598-023-33976-5

**Published:** 2023-05-09

**Authors:** Weixi Chen, Zekun Bai, Wen Bai, Wei Wang, Jiapei Guo, Mengnan Guo, Yingying Sai, Jun Shi, Jinghua Wu

**Affiliations:** 1 Tangshan Maternal and Child Health Care Hospital, North China University of Science and Technology, Tangshan, 063000 China; 2 School of Basic Medical Sciences, Peking University Health Science Center, Beijing, 100191 China; 3 Tangshan Nanhu Hospital, Tangshan, 063000 China

**Keywords:** Cancer, Cell biology, Diseases, Gastroenterology

## Abstract

Hepatocellular carcinoma (HCC) is a symptomatic disease involed multi-stage program. Here, we elucidated the molecular mechanism of LncTUG1 in the regulation of HCC evolvement. And that may in all likelyhood supply a innovative latent target for HCC’s diagnoses and prognosis. LncRNA TUG1, miR-144-3p, RRAGD and mTOR signaling pathway were screened as target genes in the database, and their expression levels at the cytological level were verified utilized qRT-PCR, Western Blot and immunohistochemistry. Then, we adopted CCK-8, Transwell and flow cytometry assays to estimate cell proliferation, invasion and apoptosis. By use of luciferase reporter assay, the relationships of LncRNA TUG1, miR-144-3p and RRAGD was confirmed. In addition, the LncRNA TUG1-miR-144-3p-RRAGD-mTOR signaling pathway in HCC cells was verified adopted rescue experiment and confirmed by xenotransplantation animal experiment. LncTUG1 in HCC tissues from three databases were identified and further verified through qRT-PCR in HCC cells (Huh7, Hep3B). Knockdown the LncTUG1 could increase apoptosis and inhibite invasion and proliferation in HCC cells. Using inhibitors and activators of the mTOR/S6K pathway, LncTUG1 was confirmed to regulate HCC progression by the mTOR/S6K pathway. Luciferase reporter assay demonstrated that TUG1 negatively regulates miR-144-3p. Furthermore, miR-144-3p negativly regulates RRAGD by way of interacting with the 3′UTR of the RRAGD mRNA in HCC utilized luciferase reporter assay. In vivo, we also discovered that neoplasm weight and tumor volume reduced significantly in subcutaneous xenograft nude mouse models derived from sh-LncTUG1-expressing Huh7 cells. And the expressions of p-mTOR, p-S6K and RRAGD were decreased obviously while the miR144-3p increased in subcutaneous xenograft nude mouse models. In a word, the research suggests that LncTUG1 targets miR-144-3p while miR-144-3p binds to RRAGD mRNA, which induces mTOR/S6K pathway activation and promotes the progression of HCC.

## Introduction

Hepatocellular carcinoma (HCC) is a common tumor in humans^1^. The development of HCC is a Multi-stage program, it was initially a symptomatic disease and ultimately leads to a late diagnosis^2–5^.Therefore, to improve the prognosis of HCC, we urgently need to identify some innovative biomarkers for early diagnostic.

Long non-coding RNAs (LncRNAs) are noncoding molecular transcripts with longtitude of over 200 nucleotides. It crucially affects genetic expression. And thus it has pivotal functions in influencing multifarious cellular procedure like the cell structure integrity, cell cycle and stem cell pluripotency^6–8^. In addition, the differential expression of LncRNA in tissues has close correlation with the mechanism of neoplasm progression^9–12^.

In recent years, many experts and scholars have paid attention to the mechanism of LncRNA in tumor. LncRNA has been proved to be a key regulator of most cell processes and cancer progress, and has been identified as a biomarker and functional regulator of human cancer prognosis^13,14^. For example, the expression level of TSLNC8 is negatively correlated with the pathological stage of tumor, and its increased expression can also inhibit the metastasis of tumor cells and the division and proliferation of tumor cells. The antitumor effect of TSLNC8 in HCC is mediated by the inactivation of IL-6/STAT3 pathway^15^. TUC339 was involved in the regulation of macrophage activation. And promotes HCC cell proliferation and reduces adhesion to extracellular matrix^16^. LncRNA-PDPK2P promotes HCC progression through PDK1/AKT/caspase 3 signaling pathway. lncRNA-PDPK2P can promote the progression of HCC, suggesting that lncrNA-PDPK2P may be a biomarker of clinical value as a molecular target for diagnosis, prognosis and treatment of hepatocellular carcinoma^17^. Hundreds of lncRNA have identified HCC-associated lncRNAs, some of these can serve as biomarkers for HCC in the aspect of diagnosis and prognosis^18^. Nevertheless, compared with the accumulating number of HCC-associated lncRNAs, only very few of them have been well-studied and have clear functions and mechanisms so far.

LncTUG1^19,20^, a recently identified oncogenic lncRNA, increasing in small cell lung cancer and inducing it’s progression^21,22^. However, the exprssion, targets and regulated pathways of LncTUG1 in HCC are still noncomprehensive.

Here, our research with the purpose of identifing the potential mechanisms, specific targets and related pathways of LncTUG1 in HCC, and explore fresh biomarkers for HCC in the aspect of diagnosis and prognosis.

## Materials and methods

### Cell culture

Cells (Huh7, Hep3B, L02) were obtained from the National Collection of Authenticated Cell Cultures (Shanghai, China). Cells culture: Dulbecco’s Modified Eagle’s Medium (DMEM, Giboc, USA), enriched with 100 μg/mL streptomycin, 100 U/mL penicillin (Solarbio Beijing, China) and 10% fetal bovine serum (BI, USA). Cells incubate: 37 °C, 5% CO_2_.

### Bioinformatics technology

There were 118, 350, 2125 differentially expressed LncRNAs in LncRNA disease public databases (http://www.cuilab.cn/LncRNAdisease), TCGA (https://portal.gdc.cancer.gov/) and GSE101728 download from the NCBI (https://www.ncbi.nlm.nih.gov/) respectively. The signaling pathways were obtained by Metascape’s GO and Pathway enrichment analysis (http://metascape.org/gp/index). LncACTdb 2.0 was used to predict the miRNA associated with TUG1, TargetScan was used to predict the miRNA combined with RRAGD 3′UTR. Furthermore, the StarBase (http://starbase.sysu.edu.cn) and Kaplan–Meier Plotter (http://kmplot.com/analysis/) were used for candidate genes’ expression, prognosis, and relationships in this study.

### Quantitative real-time PCR analysis (qRT-PCR)

Adopted Trizol reagent (Solarbio, China) to collect RNA in cells. cDNA was composed utilizing the Kit for synthesis of First-Strand cDNA (GeneCopoeia, China). qRT-PCR was implement utilizing SYBR Green qPCR Mix 2.0 (GeneCopoeia). Utilized the GAPDH mRNA as the interior control for lncRNA. For miRNA, qRT-PCR was implement utilizing the All-in-One miRNA qRT-PCR Kit (GeneCopoeia), and make use of U48 as an internal control.

### Cell transfection

Huh7 cells were seeded in 6-well plates (1 × 10^5^ cells/well). Transfection was performed when cell density reached 70–80%. sh-TUG1 (GeneCopoeia, China) MOI 1:1; overexpression miR-144-3p (OBiO, China) MOI 5:1; sh-RRAGD and overexpression RRAGD (Genecopoeia,China) MOI 5:1; si-RRAGD (Genecopoeia,China) 2.5ul/ml. Green fluorescence was observed by fluorescence microscopy (CKX53,OLYMPUS,JAPAN) to determine transfection efficiency.

### Western blot analysis

Collected cell proteins, utilized 10% SDS-PAGE to separate proteins, after that proteins were transferred to PVDF membranes (0.45 µm). Membranes were blocked with 5% skimmed milk powder solution and incubated with antibodies including GAPDH (CST), RRAGD (CST, USA), mTOR (CST), p-mTOR (CST), p70S6K (Biogot Technology, China) and p-p70S6K (Biogot Technology), at 4 °C overnight. After that, membranes were cultured with secondary antibody (Santa Cruz, USA), and ECL detection system (Bio-Rad, USA) was utilized to measure the protein bands.

### Cell Counting Kit-8 (CCK-8) assay

At 0 h, 24 h, 48 h and 96 h, 10 μl solutions (Boster, Wuhan, China) were added to 96-well plate (100 µl per well). Utilized microplate reader (Kodak, USA) to measure the absorbance (450 nm) after incubation (2 h).

### Flow cytometry

Using PBS bufffer to collect and wash cell suspension (1 × 10^6^ cells/ml) twice. Cells were stained with Huh7 PE (BD, USA), Huh7 7-ADD (BD, USA), Huh7 PE and 7-ADD together, PE and 7-ADD together. A negative control was processed in parallel. Then, measured cell apoptosis by FACSCanto II flow cytometer (Beckman, USA).

### Transwell assay

Placed 200ul cell suspension (2 × 10^5^ cells/ml, in serum-free medium) into upper chamber of a Transwell system. Added 600 μl medium containing 10% FBS to the lower chamber. 24 h of culture, cells that migrated or invaded the lower chamber were fixed and stained with 20% Giemsa. Five visions were randomly selected; stained cells numbers were counted and the average was calculated by microscope.

### Luciferase reporter assay

For luciferase assays, 293 T cells (7 × 10^4^ cells/well) were placed into 24-well plates and hatched overnight at 37 °C. Using Lipofectamine 2000 reagent, transfection was performed (1.5 µl per well) in Opti-MEM medium and Opti-MEM 500 ng, mimic 0.6 µl, when the cell density reached approximately 80–90%. Cell culture: 37 °C, 5% CO_2_, 3 days. Luciferase assays were performed using fluorescence microscopy (CKX53, OLYMPUS, JAPAN).

### Immunohistochemical (IHC) analysis

Paraffin-embedded slices of HCC tissues in model-mices were dewaxed adopting xylene, then hydrated in ethanol. The sections were heated to boiling for 15 min and then held at room temperature for 10 min. Make use of 5% BSA to block the slices for 37 °C, 20 min. Then incubated with specific primary antibody against RRAGD (CST, USA) at 4 °C overnight. Incubating with the related secondary antibodies for 37 °C, 40 min. Then the color was detected by a DAB chromogenic Kit (Wuhan, China). Images were obtained using an Olympus light microscope.

### Tumor xenograft model

Injected Huh7 cells (1 × 10^7^cells) with or without sh-LncTUG1 infection into 5-week-old Balb/c nude mices. The method was right underarm (forelimb) subcutaneously. Neoplasm volume was monitored and computed as volume (mm^3^) = 1/2 (tumor long diameter × tumor short diameter^2^). Anesthetized mice were weighed, photographed. Neoplasms were harvested. Histomorphological evaluation was performed by hematoxylin and eosin (H&E) staining.

All animal experiments were conducted in accordance with ethical permits approved by the North China University of Science and Technology Animal Care and Use Committee (2018509).

Furthermore, all animal experiments were performed in accordance with relevant guidelines and regulations and reporting in the manuscript follows the recommendations in the ARRIVE guidelines 2.0.

### Declaration

All methods, including those involving animals, were conducted in accordance to standard protocols mandated by the Institutional Review Board.

### Enrichment analysis of the metascape

Metascape is a website tool that integrates more than 40 bioinformatic databases, providing easy access to comprehensive data analysis through one-click quick analysis, including biological pathway enrichment analysis, protein interaction network structure analysis and rich gene annotation capabilities, and presents the results in an easy-to-understand, high-quality graphical language. First, open Metascape's homepage (http://metascape.org/gp/index), click Express Analysis to upload or paste the gene list. Next, submit the species source of gene list and which species you want to use as a benchmark. We input the species as H sapiens (394). Then, in the Enrichment option of Custome Analysis, from the bottom to the top, select the GO or KEGG related database and check pick selective, click Enrichment Analysis.

### Statistical analysis

Datas were analyzed utilizing SPSS17.0 software (IBM, NY, USA). T-test was used. Make use of Kaplan–Meier method to determine Overall survival curves. *P* < 0.05 was supposed to manifest statistical significance.

### Ethical approval

This study was approved and consented by the ethics committee of the Affiliated Hospital of North China University of Science and Technology.

## Results

### LncTUG1 identification and its regulation of HCC progression in vitro

**Figure 1:**
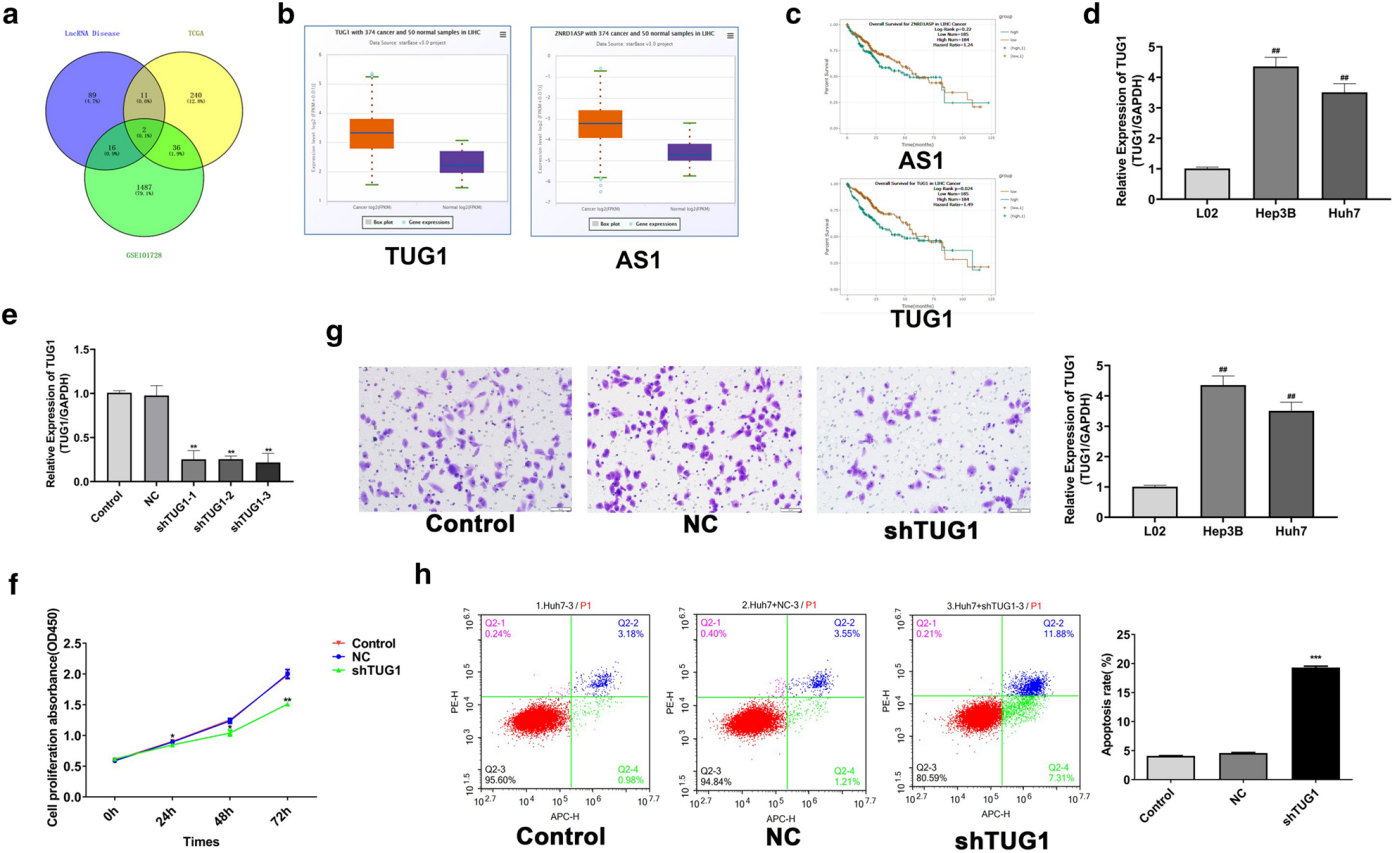
Screening of the LncTUG1 and its regulation of HCC progression in vitro. Two lncRNAs in HCC tissues from three databases were identified through Venn diagram analysis (**a**). The expressions of LncTUG1 and ZNRD1-ASP in HCC were determined using StarBase (**b**). HCC patient prognosis was evaluated utilized Kaplan–Meier analysis (**c**). qRT-PCR detected the LncTUG1 expression in cells (**d**). Knockdown efficiency of shTUG1 was confirmed via qRT-PCR (**e**). CCK-8 (**f**), flow cytometry (**g**) and Transwell assays (**h**) determined the cell proliferation, apoptosis and invasion levels in Huh7 cells. Repeated each experiments for three times, results were homologous. *P < 0.05 versus NC group; **P < 0.01 versus NC group; ***P < 0.001 versus NC group; ^##^*P* < 0.01 versus control cells.

To sieving the differentially expressed lncRNAs in HCC, we used the NCBI, TCGA and LncRNA disease public databases (http://www.cuilab.cn/LncRNAdisease). Venn diagram analysis identified two differentially expressed lncRNAs in the three databases: LncTUG1 and ZnRD1-ASP (Fig. 1a). The expression of LncTUG1 and ZnRD1-ASP were measured in HCC tissues by way of StarBase database (http://starbase.sysu.edu.cn), and the results displayed that compared with normal tissues, LncTUG1 and ZnRD1-ASP expressions were both rised in HCC tissues (Fig. 1b). In addition, the higher LncTUG1 expression, the worse prognosis of sufferers (*P* < 0.05), while ZNRD1 was not (*P* > 0.05) (Fig. 1c). We verified high expression of LncTUG1 in HCC cells utilized qRT-PCR (Fig. 1d).

In order to find out the act of LncTUG1 on HCC, LncTUG1 was knocked down in Huh7 cells by shRNA, and the capacities of proliferation, invasion and apoptosis were measured adopting CCK-8, Transwell and flow cytometry assays severally. Results displayed that in Huh7 cells with TUG1 knockdown, the invasion and proliferation capacity reduced evidently and the level of apoptosis rased (Fig. 1e–h). These findings revealed that LncRNA TUG1 is overexpressed in HCC and was relevent to the evolvement of hepatocellular carcinoma.

### LncTUG1 activated the mTOR/S6K pathway and regulated HCC cells malignant behavior

**Figure 2:**
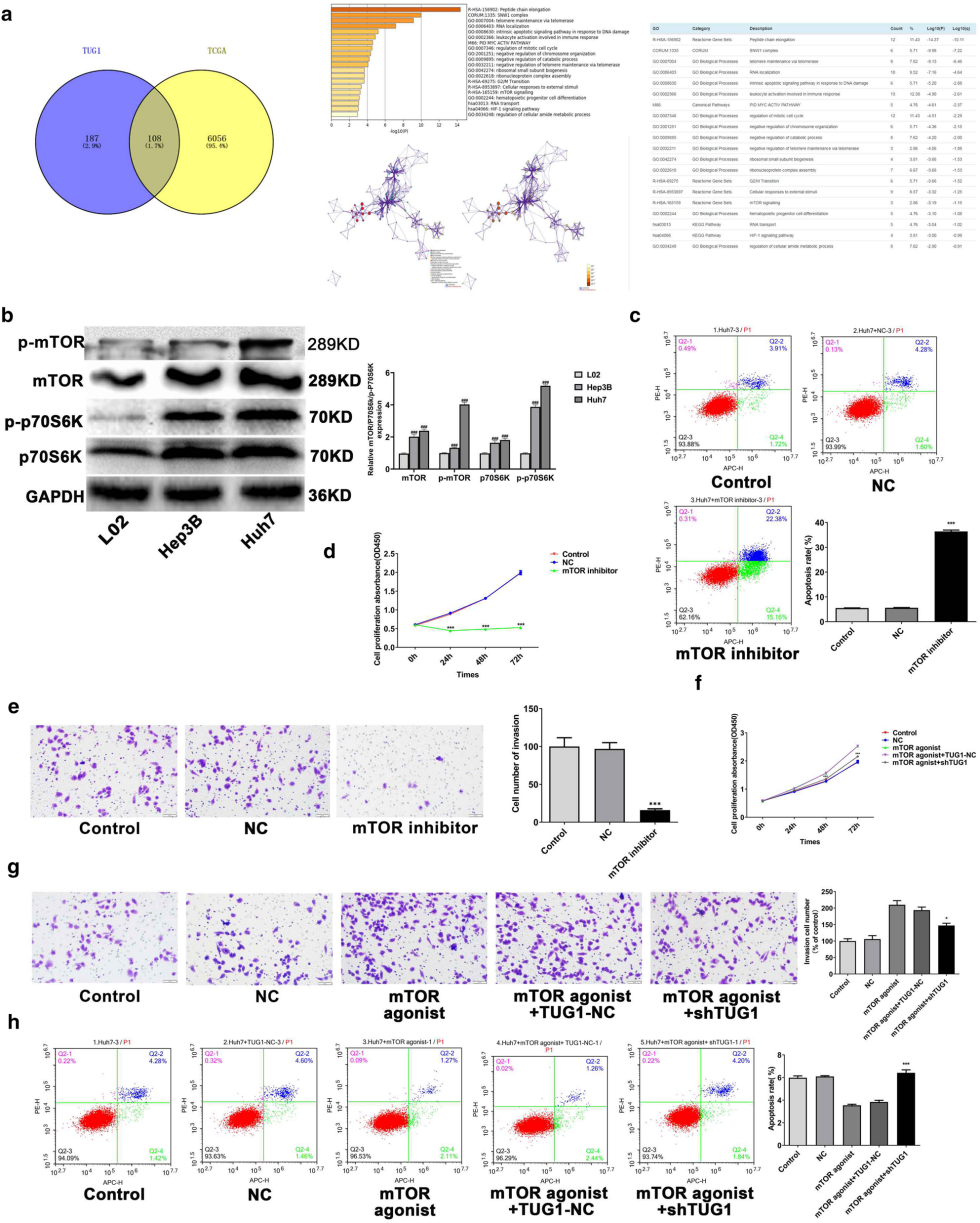
LncTUG1 activated the mTOR/S6K pathway and regulated HCC cells malignant behavior. mTOR pathway was selected through GO and pathway enrichment analysis in Metascape (**a**). mTOR, p-mTOR, p70S6K and p-p70S6K were detected in HCC cells by western blot (**b**). The apoptosis (**c**), proliferation (**d**) and invasion (**e**) of Huh7 cells treated with mTOR inhibitor were evaluated by flow cytometry, CCK-8 and Transwell assays, respectively. Adopted CCK-8, Transwell and flow cytometry assays to measure the proliferation (**f**), invasion (**g**) and apoptosis (**h**) of shTUG1-expressing Huh7 cells treated with the mTOR agonist. Repeated each experiments for three times, results were homologous. ^###^*P* < 0.001 versus control cells; ^***^P < 0.001 versus NC group; ^*^P < 0.05 versus mTOR agonist + TUG1-NC group.

To examine the mechanism by which LncTUG1 may promote HCC progression, we first selected genes related with LncTUG1 and make GO enrichment analysis in StarBase (http://starbase.sysu.edu.cn) and Metascape (http://metascape.org/gp/index). On account of it remarkably affects the evolement of HCC^23–25^, the mTOR/S6K pathway was selected for study (Fig. 2a). In order to confirm the activation of mTOR/S6K pathway in HCC cells, we examined the p-mTOR and p-S6K levels by western blot. The p-p70S6K and p-mTOR expressions were both obviously increased in HCC cells (Fig. 2b). Next, we used mTOR inhibitor or agonist to treat Huh7 cells and measured the levels of apoptosis, proliferation and invasion by Transwell, CCK-8 and flow cytometry, respectively. When cells treated with the inhibitor, the apoptosis level increased significantly, and the proliferation and invasion decreased dramatically. In the meantime, the agonist displayed opposite results (Fig. 2c–e).

Next, we further to confirm the regulation of LncTUG1 to mTOR/S6K. In cells with LncTUG1 knockdown, addition of the mTOR agonist can lead to reduced cell proliferation and invasiveness while increased the level of cell apoptosis (Fig. 2f–h). The above results indicated that LncTUG1 regulates cell proliferation, apoptosis and invasion via the mTOR/S6K pathway in HCC.

### LncTUG1 regulates the progression of HCC through activated RRAGD-mTOR/S6K pathway

**Figure 3:**
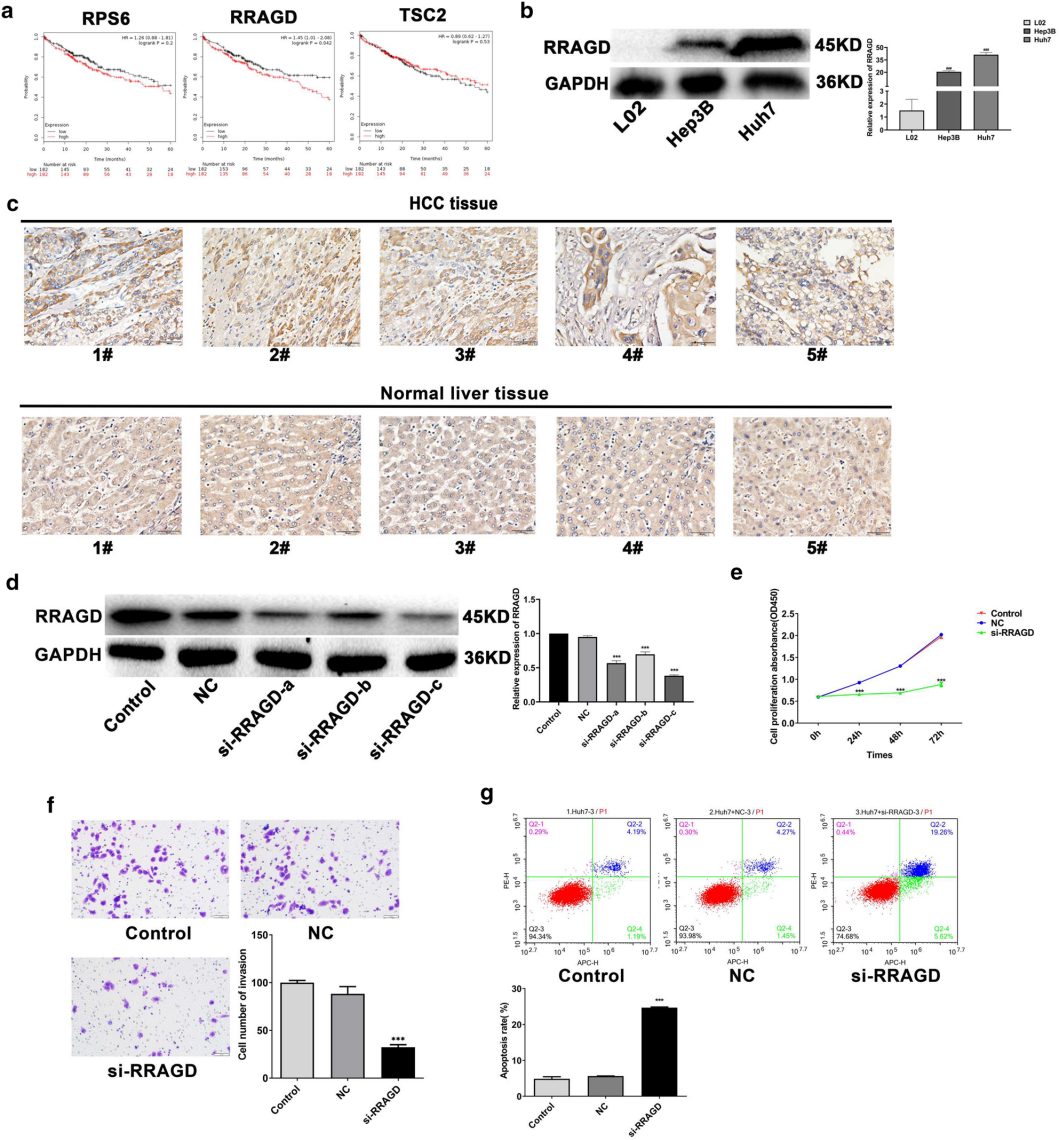
Effects of RRAGD on the malignant biological behaviors of HCC cells. The prognosis of HCC sufferers was estimated adopted Kaplan–Meier analysis (**a**). RRAGD expression in Huh7, Hep3B and L02 cells was determined through western blot (**b**). IHC was sed to detect RRAGD expression in HCC tissues (**c**). Utilized western blot to measure the knockdown efficiency of si-RRAGD (**d**). The proliferation (**e**), invasion (**f**) and apoptosis (**g**) of si-RRAGD-transfected Huh7 cells through CCK-8, Transwell and flow cytometry assays respectively. Repeated each experiments for three times, results were homologous. ***P < 0.001 versus NC group; ^###^*P* < 0.001 versus control cells.

To more closely examine the mechanism by which LncTUG1 regulates the mTOR/S6K pathway, we examined genes related to mTOR pathway and genes regulated by LncTUG1 in HCC and obtained three genes. We analyzed the association of the three genes on HCC patient prognosis by Kaplan–Meier Plotter (http://kmplot.com/analysis/). Results displayed that high RRAGD expression is relevent to the poor prognosis (*P* < 0.05) while not in patients of high RPS6 or TSC2 expression; furthermore, high RRAGD expressions were observed in HCC tissues and cells (Fig. 3a–c).

RRAGD, a GTP binding protein, functions as a molecular switch through mTOR pathway^26^. To study the role of RRAGD in HCC, siRNA was used to knockdown RRAGD. Knockdown of RRAGD repressed cell proliferation and invasion (Fig. 3d–f), while apoptosis level increased (Fig. 3g).

**Figure 4:**
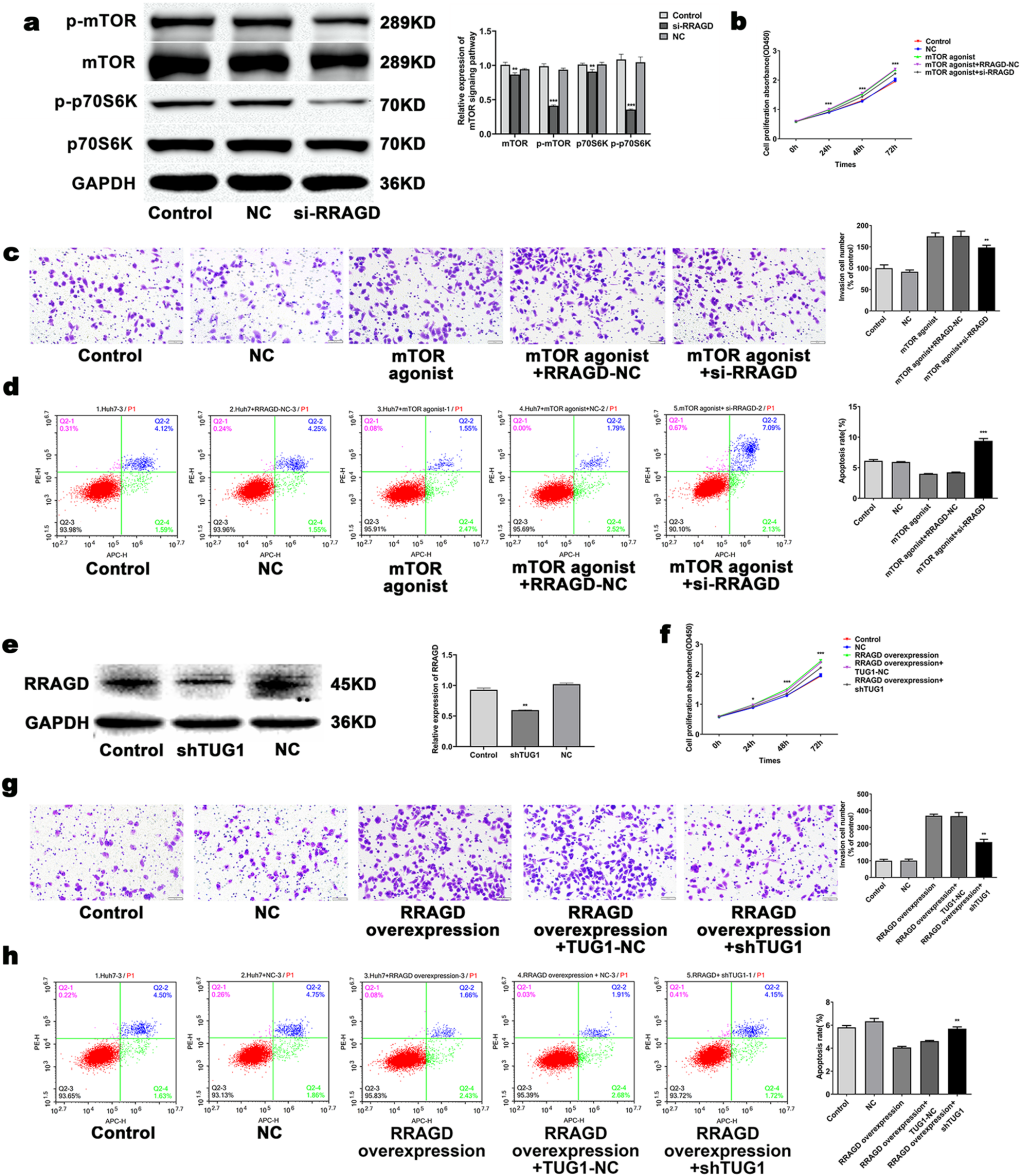
LncTUG1 regulates the progression of HCC by the RRAGD-mTOR/S6K pathway. Western blot analysis was performed in Huh7 cells for the indicated proteins (**a**). Adopted CCK-8, Transwell and flow cytometry assays to evaluated the proliferation (**b**), invasion (**c**) and apoptosis (**d**) of si-RRAGD Huh7 cells treated with mTOR agonist. The expression of RRAGD in shTUG1-transfected Huh7 cells was tested via western blot (**e**). The proliferation (**f**), invasion (**g**) and apoptosis (**h**) of RRAGD-overexpressing Huh7 cells transfected with sh-LncTUG1 were respectively evaluated by means of CCK-8, Transwell and flow cytometry assays. Repeated each experiments for three times, results were homologous. *P < 0.05 versus the NC group; **P < 0.01 versus NC group; ***P < 0.001 versus NC group.

Results of Western blot displayed that RRAGD activates the mTOR/S6K pathway (Fig. 4a). We activated the mTOR pathway by mTOR agonist in cells transfected with si-RRAGD. The results showed that addition of mTOR agonists reversed the effects of cell proliferation, apoptosis, and invasion produced by the downregulation of RRAGD (Fig. 4b–d).

We examined RRAGD after knockdown of TUG1 and found that the level of RRAGD decreased significantly after TUG1 knockdown (Fig. 4e). After knocking down LncTUG1 in Huh7 cells, the proliferation and invasion were inhibited and apoptosis increased which can be reversed by the overexpressed RRAGD (Fig. 4f–h). These results showed that LncTUG1 activates the mTOR/S6K pathway in HCC cells by upregulating RRAGD, and promotes the progression of HCC.

### LncTUG1 is involved in HCC progression by directly targeting miR-144-3p in Huh7 cells

**Figure 5:**
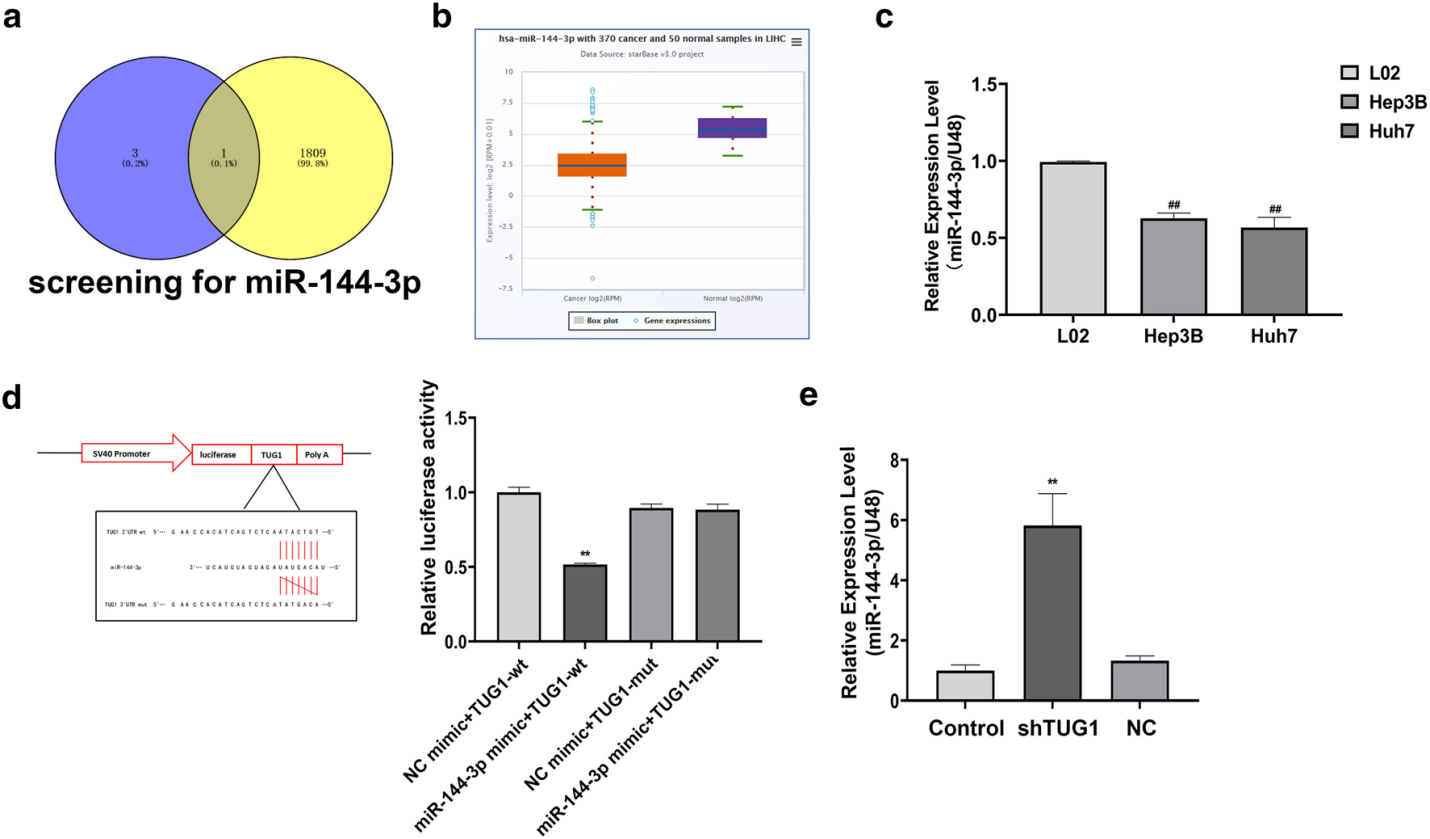
miR-144-3p was down-regulated by TUG1 in HCC cells. miR-144-3p was screened in the LncACTdb2.0 and TargetScan databases (**a**). miR-144-3p expression in HCC was verified utilized StarBase (**b**). Utilized qRT-PCR to measured miR-144-3p expression in cells (Huh7, Hep3B, L02) (**c**). Adopted Luciferase reporter gene assays to determine the relationship between miR-144-3p and LncTUG1 (**d**). Adopted qRT-PCR to determined miR-144-3p expression in sh-LncTUG1-expressing Huh7 cells (**e**). Repeated each experiments for three times, results were homologous. ^##^*P* < 0.01 versus control cells. **P < 0.01 versus NC group.

Our results above suggest that LncTUG1 can regulate HCC progression through RRAGD, but the specific mechanism is unclear. To more closely explore the mechanism of LncTUG1 in HCC, we examined potential target miRNAs of LncTUG1 in the LncACTdb2.0 (http://www.bio-bigdata.net/LncACTdb/index.html) database. We identified four miRNAs associated with TUG1 in HCC: miR-29a-3p, miR-29b-3p, miR-29c-3p and miR-144-3p. In addition, prediction by TargetScan (http://www.targetscan.org/vert_71/) revealed 1810 miRNAs may bind to the 3′UTR of RRAGD mRNA. In conclusion, miR-144-3p is the only common miRNA in these results (Fig. 5a).

The analysis of miR-144-3p utilized StarBase (http://starbase.sysu.edu.cn) revealed that compared with normal samples, miR-144-3p expression was dramatically repressed in HCC (Fig. 5b). In addition, qRT-PCR results displayed that the miR-144-3p mRNA significantly reduced in cells (Fig. 5c).

Next, we utilized luciferase reporter assays to elucidate the regulatory relationship between LncTUG1 and miR-144-3p. Results anounced that miR-144-3p was the target molecular of LncTUG1 (Fig. 5d). miR-144-3p expression rised in LncTUG1-knockdown cells (Fig. 5e). These results suggested a negative regulatory relationship between TUG1 and miR-144-3p.

**Figure 6:**
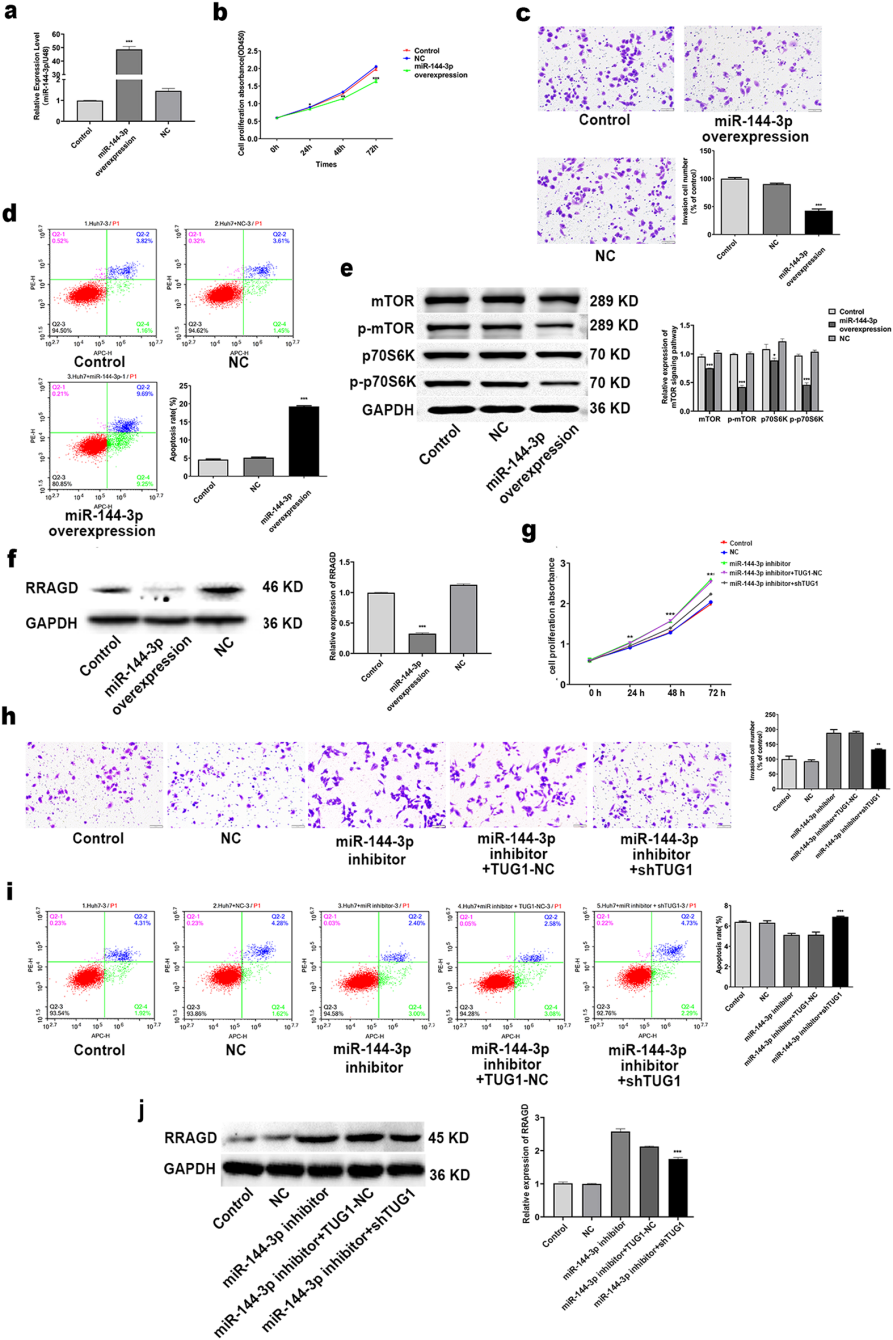
LncTUG1 is involved in HCC progression by directly targeting miR-144-3p in Huh7 cells. Adopted qRT-PCR to determine the efficiency of overexpressing miR-144-3p (**a**). The proliferation (**b**), invasion (**c**) and apoptosis (**d**) of miR-144-3p-overexpressing Huh7 cells were respectively evaluated through CCK-8, Transwell and flow cytometry assays. mTOR, p-mTOR, p70S6K, and p-p70S6K were examined in miR-144-3p-overexpressing Huh7 cells by western blot (**e**). Adopted western blot to measured RRAGD expression in miR-144-3p-overexpressing Huh7 cells (**f**). Proliferation (**g**), invasion (**h**) and apoptosis (**i**) of sh-LncTUG1-expressing Huh7 cells dealed with miR-144-3p inhibitor were determined utilized CCK-8, Transwell and flow cytometry assays. RRAGD expression in sh-LncTUG1-treated Huh7 cells dealed with miR-144-3p inhibitor was evalued through western blot (**j**). Repeated each experiments for three times, results were homologous. *P < 0.05 versus NC group; **P < 0.01 versus NC group; ***P < 0.001 versus NC group.

In order to elucidate how miR-144-3p effects on HCC, we overexpressed miR-144-3p in Huh7 cells. Overexpressed-miR-144-3p depressed the proliferation and invasion while apoptosis level increased (Fig. 6a–d). Overexpression of miR-144-3p also remarkably reduced activation of the mTOR/S6K pathway and RRAGD expression (Fig. 6e–f).

So as to expound how LncTUG1 affects miR-144-3p on cell proliferation, invasion and apoptosis, rescue experiments were performed. LncTUG1 in HCC cells attenuated the positive effect of the miR-144-3p inhibitor on cell proliferation and invasion. Cell apoptosis was inhibited by miR-144-3p inhibitor; however, these effects were eliminated by knockdown of LncTUG1 in Huh7 cells (Fig. 6g–i). Moreover, knockdown of LncTUG1 reversed the RRAGD expression induced via miR-144-3p inhibitors (Fig. 6j). Those ablve results announced that TUG1 accelerates cell proliferation, invasion while inhibits apoptosis by means of targeting miR-144-3p in HCC.

### miR-144-3p regulated HCC progression through targeting RRAGD mRNA 3′-UTR

**Figure 7:**
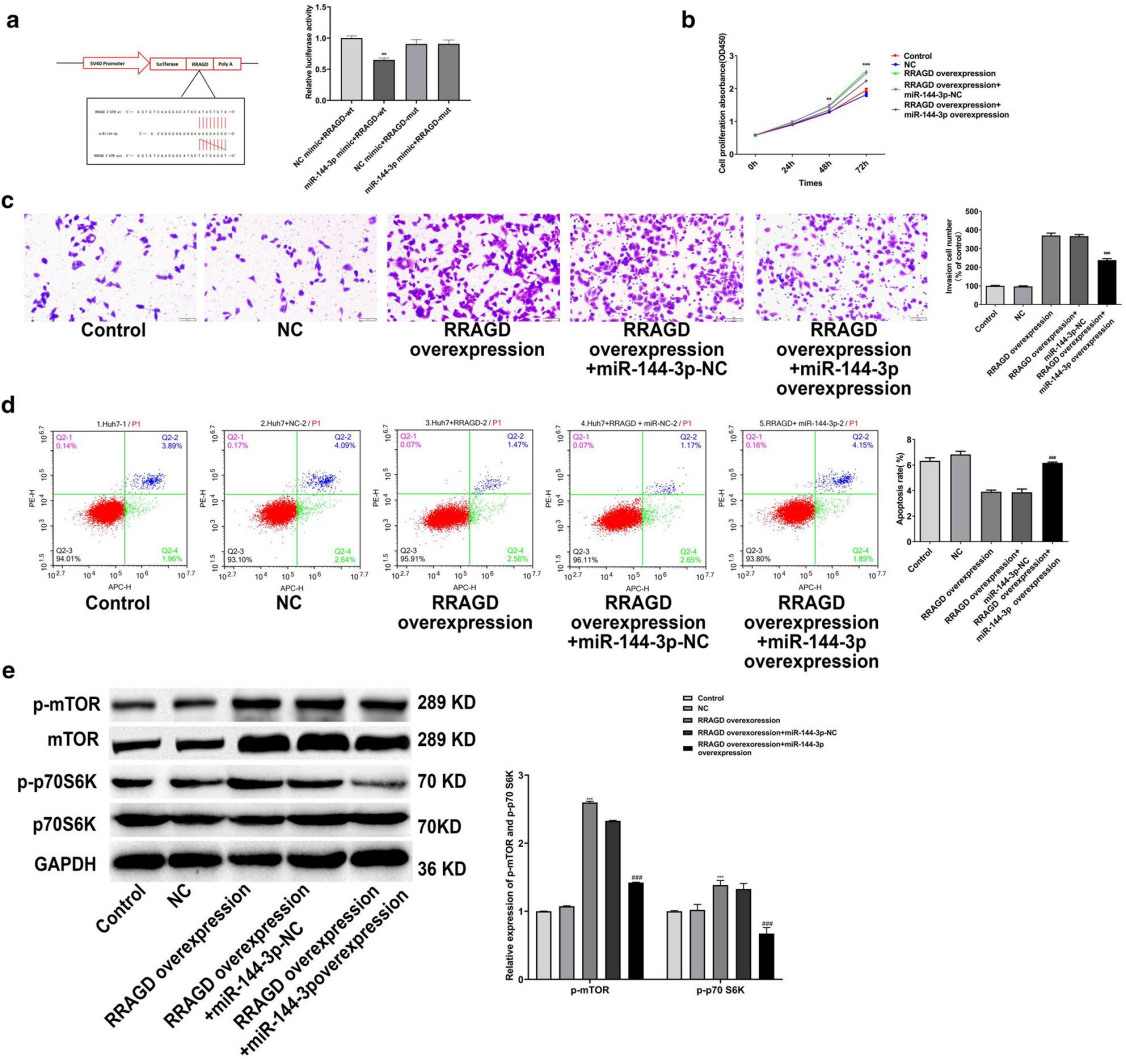
miR-144-3p regulated HCC progression through targeting RRAGD mRNA 3′-UTR. Luciferase assays were performed in HEK293T cells to determine the regulatory relationship between RRAGD and miR-144-3p (**a**). Proliferation (**b**), invasion (**c**) and apoptosis (**d**) of miR-144-3p-overexpressing Huh7 cells transfected with the RRAGD overexpression construct were respectively measured through CCK-8, transwell and flow cytometry assays. mTOR, p-mTOR, p70S6K, and p-p70S6K in miR-144-3p-overexpressing Huh7 cells transfected with the RRAGD overexpression construct were evalued via western blot (**e**). Repeated each experiments for three times, results were homologous. **P < 0.01 versus NC group; ***P < 0.001 versus NC group.

Whether RRAGD is the target gene of miR-144-3p was proved utilizing the luciferase reporter assay (Fig. 7a). Next we examined whether the neoplasm inhibitory effect of miR-144-3p involves RRAGD. Results announced that miR-144-3p could reverse the effects of RRAGD on cell proliferation, invasion and apoptosis (Fig. 7b–d). Furthermore, Overexpressed-miR-144-3p reversed the RRAGD-induced activation of mTOR/S6K pathway (Fig. 7e).

### LncTUG1 promotes the development of HCC in vivo through miR-144-3p/RRAGD-mTOR/S6K pathway

**Figure 8:**
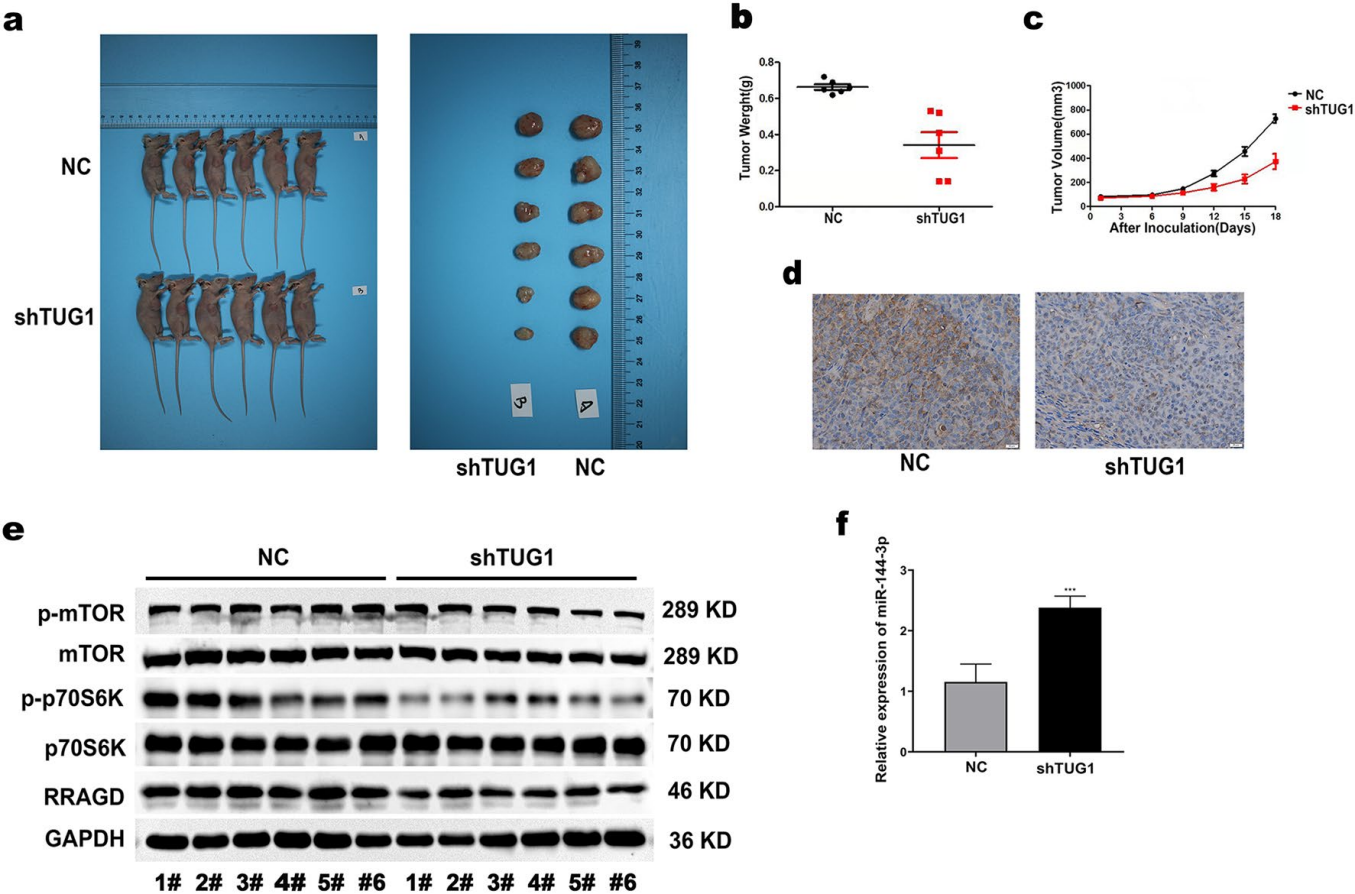
LncTUG1 promotes the progression of HCC in vivo. Nude mices were subcutaneously injected into Huh7 cells that stably transfected with sh-LncTUG1 (n = 6) or NC (n = 6) (**a**). Weight (**b**) and volume (**c**) of tumor that obtained were measured in subcutaneous xenograft nude mouse models derived from sh-LncTUG1-expressing Huh7 cells. RRAGD expression was detected by immunostaining of xenografts from mice in the sh-LncTUG1-expressing group (**d**). Evaluated mTOR, p-mTOR, p70S6K, and p-p70S6K in subcutaneous xenograft nude mouse models derived from sh-LncTUG1-expressing Huh7 cells (**e**). miR-144-3p expression was detected in subcutaneous xenograft nude mouse models derived from sh-LncTUG1-expressing Huh7 cells by qRT-PCR (**f**). Repeated each experiments for three times, results were homologous. ***P < 0.001 versus NC group.

In order to elucidate the effect of LncTUG1 on HCC progression in vivo, we evaluated tumor formation in nude mice. Mices were injected into sh-TUG1-expressing Huh7 cells to construct tumor model in vitro. Results proved that tumor volume was evidently reduced (Fig. 8a–c). In addition, IHC staining certificated that the RRAGD expression decreased in neoplasms from mice injected with sh-TUG1-expressing Huh7 cells (Fig. 8d). Western blot and qRT-PCR results announced that RRAGD expression and the activation of mTOR/S6K were significantly reduced and miR-144-3p was increased in subcutaneous xenograft nude mouse models derived from sh-LncTUG1-expressing Huh7 cells (Fig. 8e–f). These results suggest that LncTUG1 facilitates the HCC tumor growth in vivo, and these effects may occur via its regulation of the miR-144-3p/RRAGD-mTOR/S6K pathway.

## Discussion

LncRNA are key regulators of cancer progression and play roles as biomarkers for human cancer prognosis^27^. Recently, the importance of LncRNA in HCC carcinogenesis has been gradually coming to light^9,10^. By means of bioinformatics analysis, it is demonstrated that LncTUG1 expression was markedly increased in HCC tissues and cells.

High expression of LncTUG1 was positively in connection with the poor prognosis in HCC sufferers and in vitro. Besides, vivo experiments demonstrated LncTUG1 regulated cell proliferation and metastasis. Our findings manifest that LncTUG1 crucially affects the regulation of HCC neoplasm proliferation and metastasis.

The imbalance of energy metabolism momentously affects neoplasm progression. The mTOR pathway, a main pathway of energy metabolism, is aberrantly activated in tumors, and it is also involved in the evolvement of many tumors, including HCC^28^. Yet the molecular mechanisms that LncRNAs target the mTOR/S6K pathway on inducing tumor progression in HCC have not been clarified. From our results, we first screening the LncTUG1 as an activator of the mTOR/S6K pathway in HCC cells. And then, we propose that LncTUG1 can promote HCC progression via regulating the mTOR/S6K pathwaywhich has been demonstrated in vitro. Next, we firstly screened and identified the RRAGD as a key molecule to activate mTOR/S6K pathway, meanwhile an effector of LncTUG1. RRAGD, a GTP binding protein, is involved in the regulating the metabolism of amino acids, proteins and sugars. RRAGD functions as a molecular switch through mTOR pathway^26^. In view that miRNA was the main effectors of LncRNA, we then examined target miRNAs of LncTUG1 in databases and identified miR-144-3p. Studies manifested that miR-144-3p is anomalously expressed in multiple neoplasms, for instance, renal clear cell carcinoma, colorectal cancer, etc^29–33^. Our research displayed that miR-144-3p expression reduced in HCC, and induced the neoplasm progression. Besides, it is manifested that miR-144-3p directly bind to LncTUG1 at the recognition site of miRNA, and miR-144-3p was negatively correlated with LncTUG1. Meantime, luciferase reporter assays also revealed that RRAGD mRNA was another target spot of miR-144-3p. From animal experiments, when LncTUG1 was knocked down, miR-144-3p increases while the expression of RRAGD, mTOR and S6K reduced in neoplasm tissues, and the neoplasm volume is also reduced. These results confirmed all our conclusions above in vivo.

All in all, the research demonstrates LncRNA TUG1 which was up-regulated in HCC and associated with poor prognosis of HCC patients can facilitated HCC proliferation and progression, while suppressed apoptosis and targeted miR-144-3p which functioned as tumor suppressor in HCC. As a target gene of miR-144-3p, RRAGD was over-expressed in HCC and participating in the HCC process by activating the mTOR signaling pathway which suggests LncRNA TUG1 and RRAGD expression should be considered as an undesired feature of HCC and other tumors. In addition, we innovatively discovered the significance of interaction among LncRNA TUG1, miR-144-3p, RRAGD and mTOR pathway. These findings suggest that LncRNA TUG1/miR-144-3p/RRAGD/mTOR axis has a hand in the evovement of HCC and might serve a potential therapeutic target and provide novel biomarkers for HCC in the aspect of precise prevention, diagnosis and prognosis. And further clinical analysis of RRAGD is likely to provide more data support for the above conclusions, which needs to be verified in our future work.

## Conclusions

LncTUG1 targets miR-144-3p and miR-144-3p binds to RRAGD mRNA, which induces mTOR/S6K pathway activation and promotes the progression of HCC (Supplementary File 1).

## Supplementary Information

Supplementary Information.

## Data Availability

Data on the results of this study are available from the corresponding author in the light of rational request.

## Abbreviations

HCC: Hepatocellular carcinoma
RRAGD: Ras-related GTP-binding protein D
mTOR: Mannalian target of rapamycin
mTORC1: Mannalian target of rapamycin complex 1
3′-UTR: 3′-Untranslated region
DEGs: Differentially expressed genes
LncRNA: Long non-coding RNA
DEPC: Diethylpyrocarbonate
DMSO: Dimethyl sulfoxide
PBS: Phosphate buffer saline
kDa: Kilodalton
EGFP: Enhanced green fluorescent protein
PAGE: Polyacrylamide gel electrophoresis
One way-ANOVA: One-way analysis of variance
Bcl-2: B-cell lymphoma-2
TUG1: Taurine upregulation gene 1
TACE: Transarterial chemoembolization
GAPDH: Glyceraldehyde phosphate dehydrogenase
qRT-PCR: Quantitative real-time PCR
shRNA: Short hairpin RNA
NC: Negative control
