# Supplementary material for: LncTUG1 contributes to the progression of hepatocellular carcinoma via the miR-144-3p/RRAGD axis and mTOR/S6K pathway
Source: Sci Rep. 2023 May 9;13:7500. doi: 10.1038/s41598-023-33976-5 (PMC10170139; doi:10.1038/s41598-023-33976-5)

Figure2-b-WB(GAPDH)

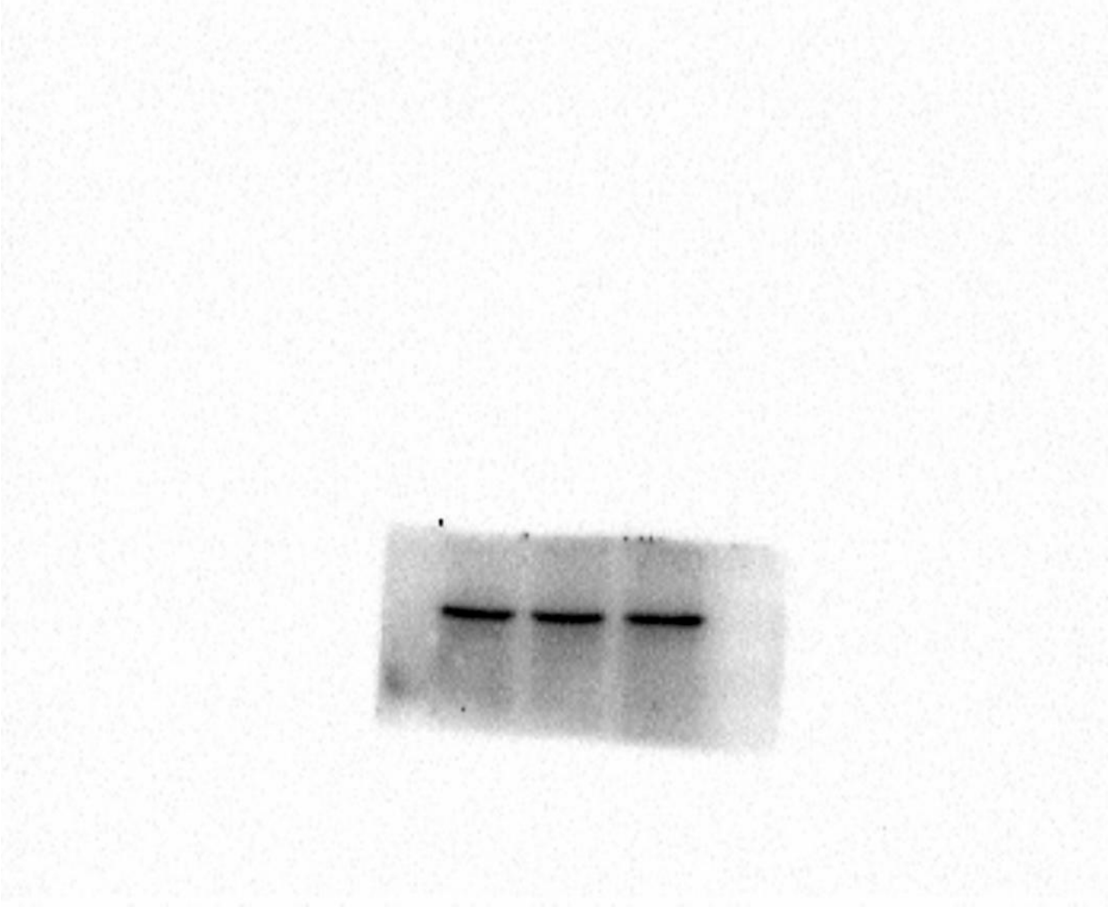

Figure2-b-WB(mTOR)

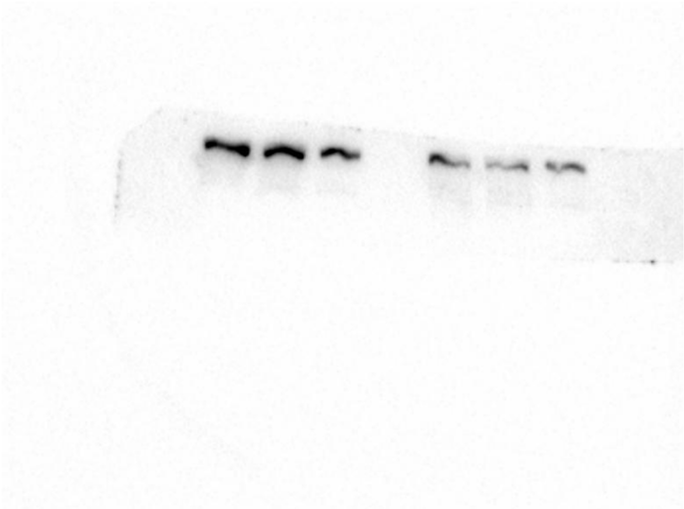

Figure2-b-WB(Figure70S6K)

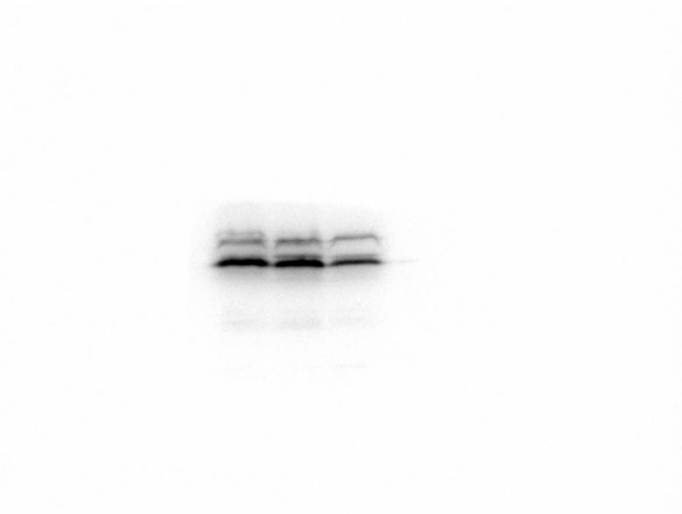

Figure2-b-WB(p-mtor)

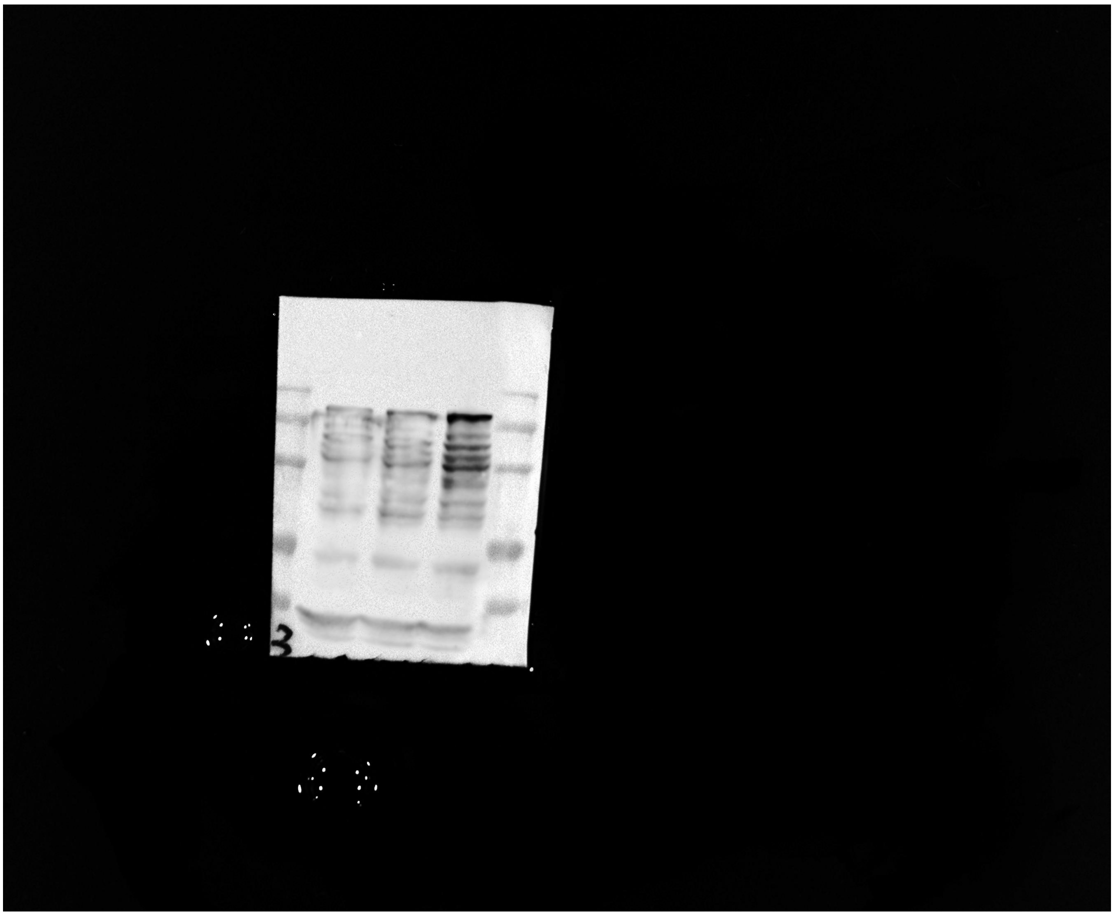

Figure2-b-WB(p-Figure70S6K)

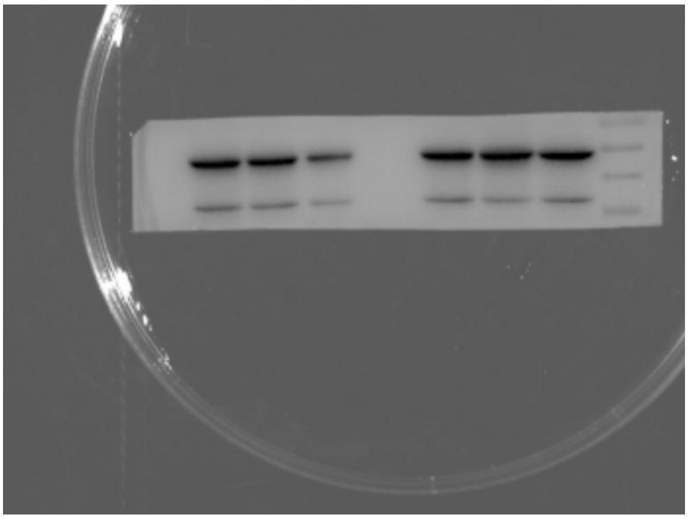

Figure3-b-WB(GAPDH)

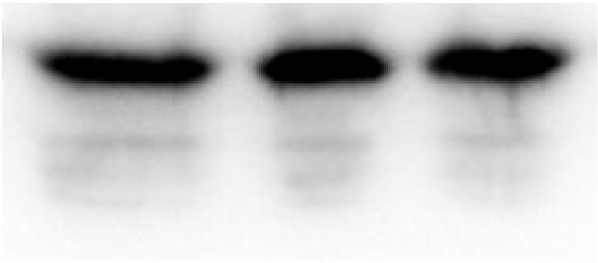

Figure3-b-WB(RRAGD)

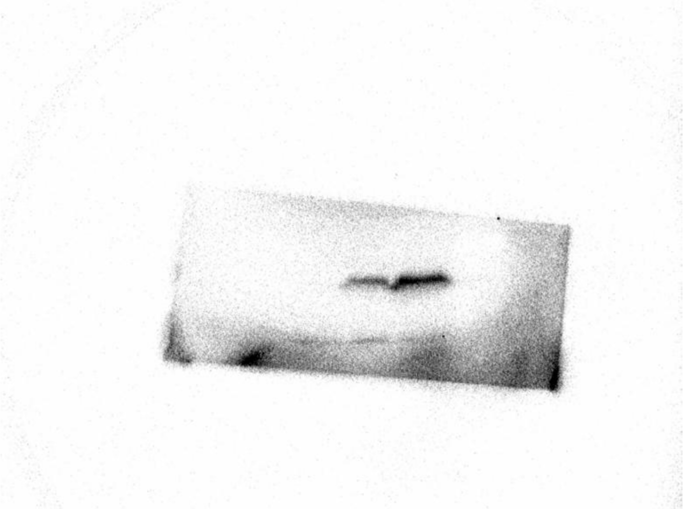

Figure3-d-WB(GAPDH)

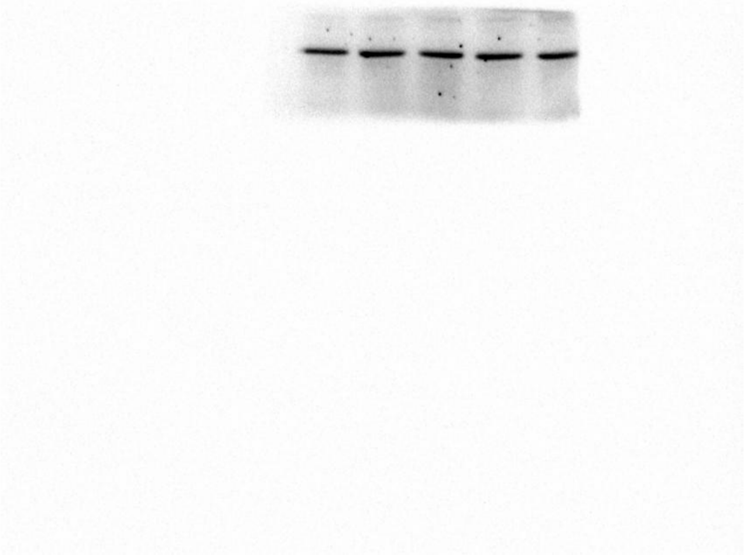

Figure3-d-WB(siRRAGD)(above)

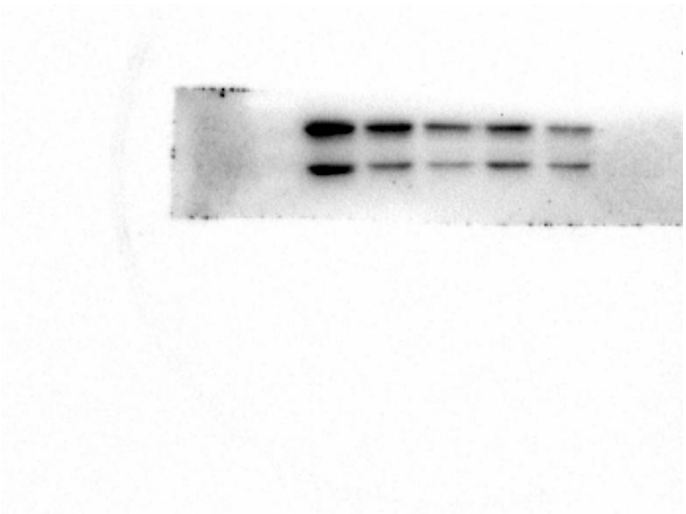

Figure4-a-WB(GAPDH)

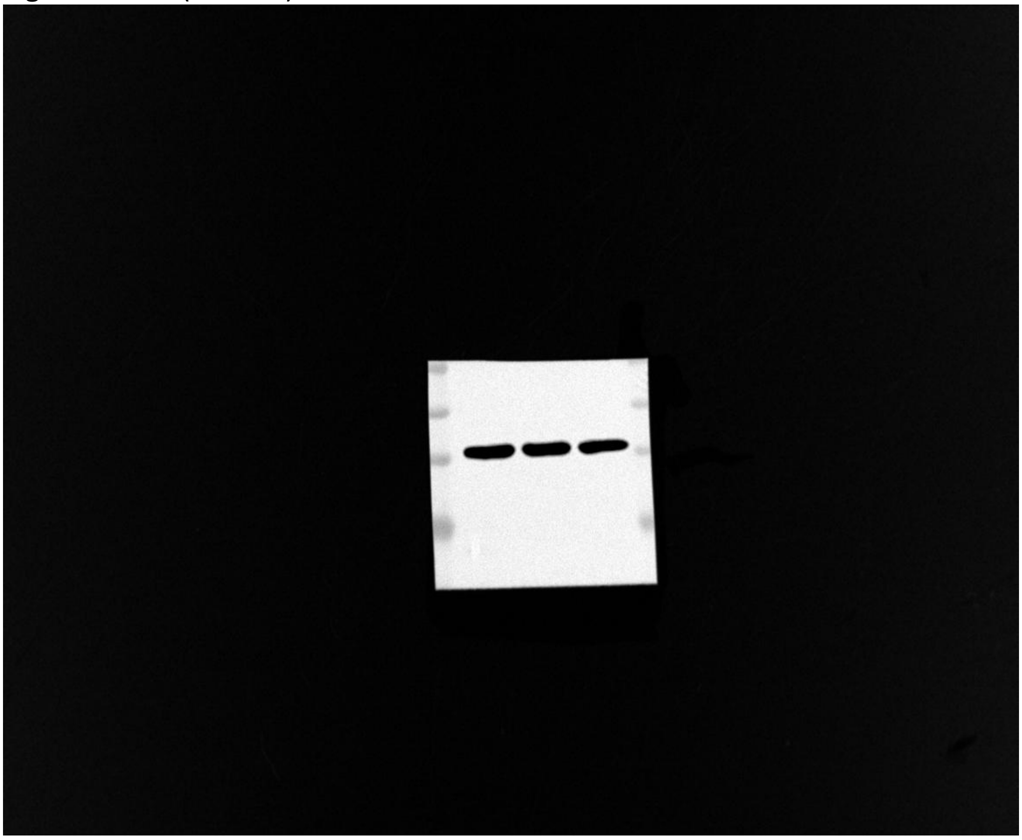

Figure4-a-WB(mTOR)

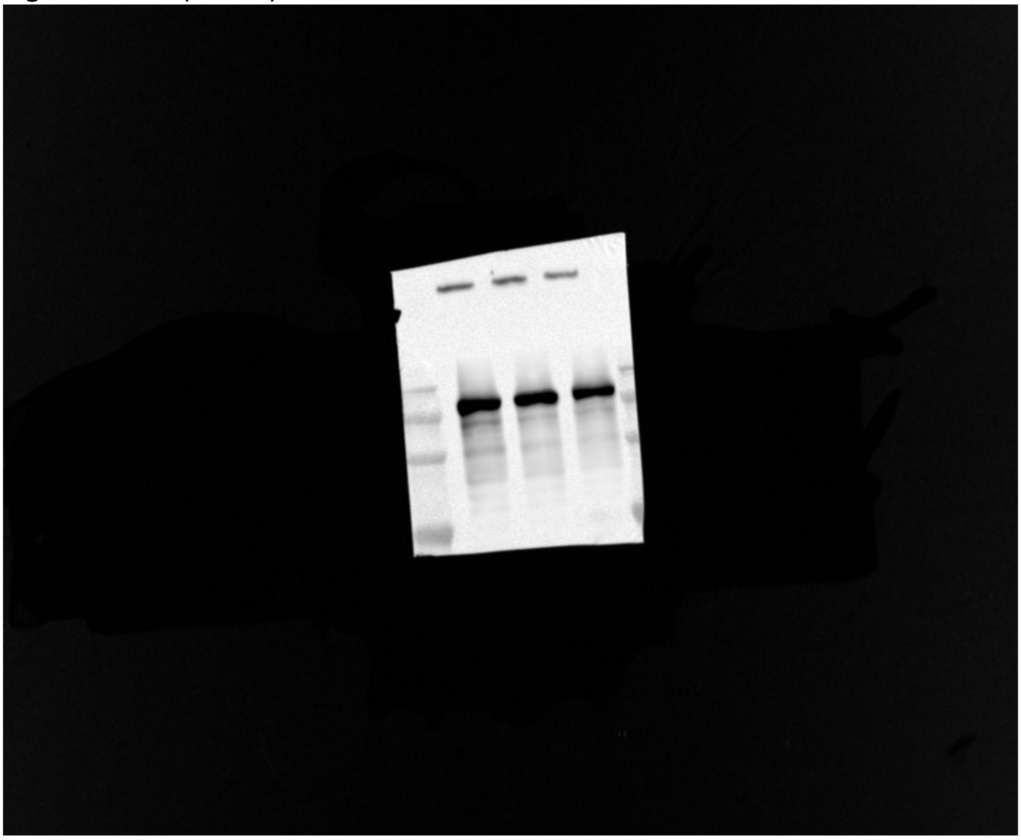

Figure4-a-WB(p-mTOR)

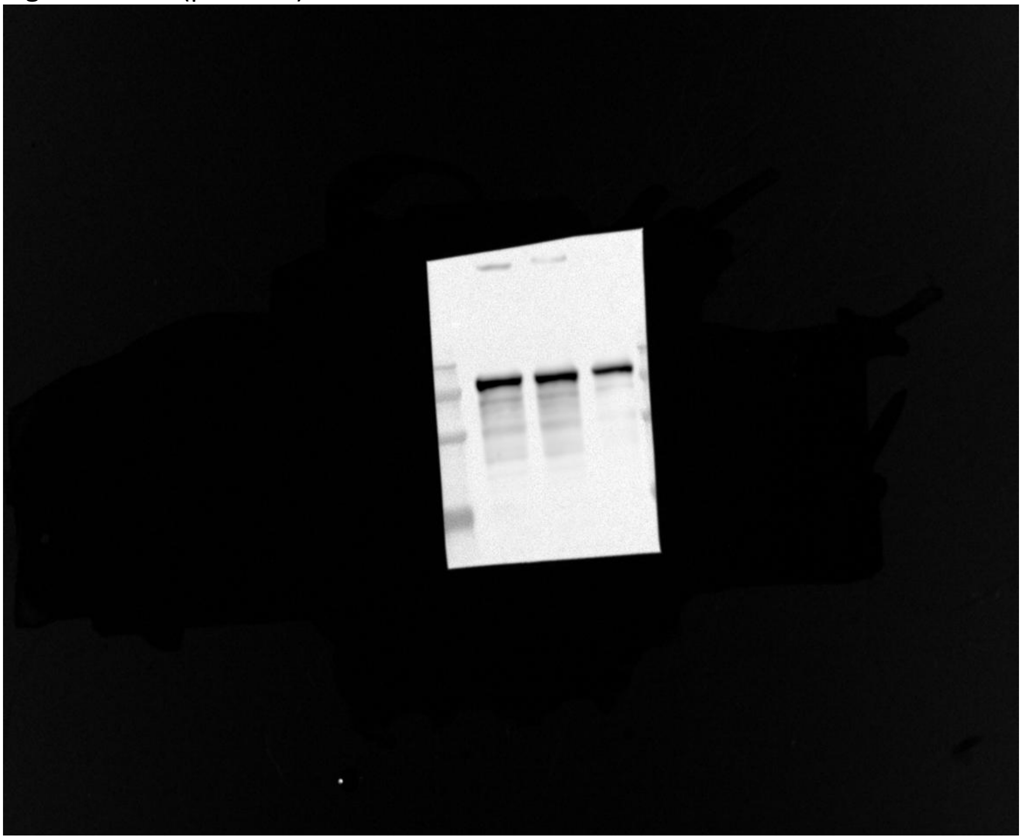

Figure4-a-WB(p-Figure70S6K)

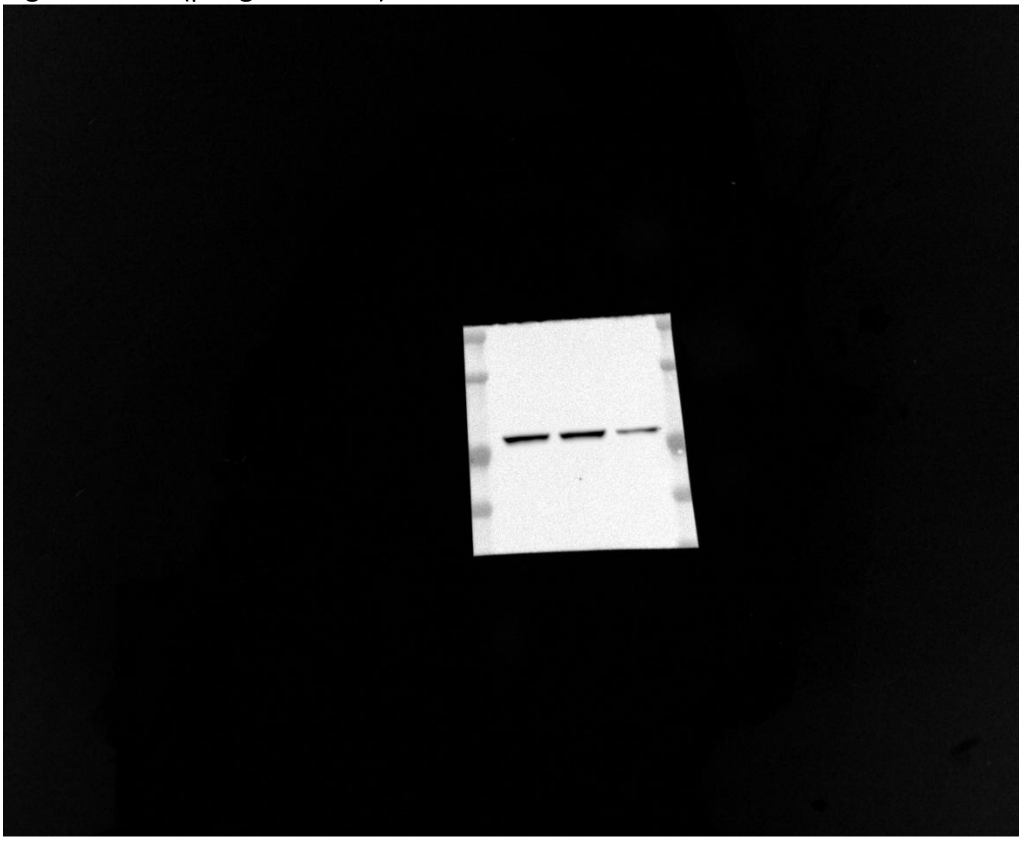

Figure4-a-WB(S6K)

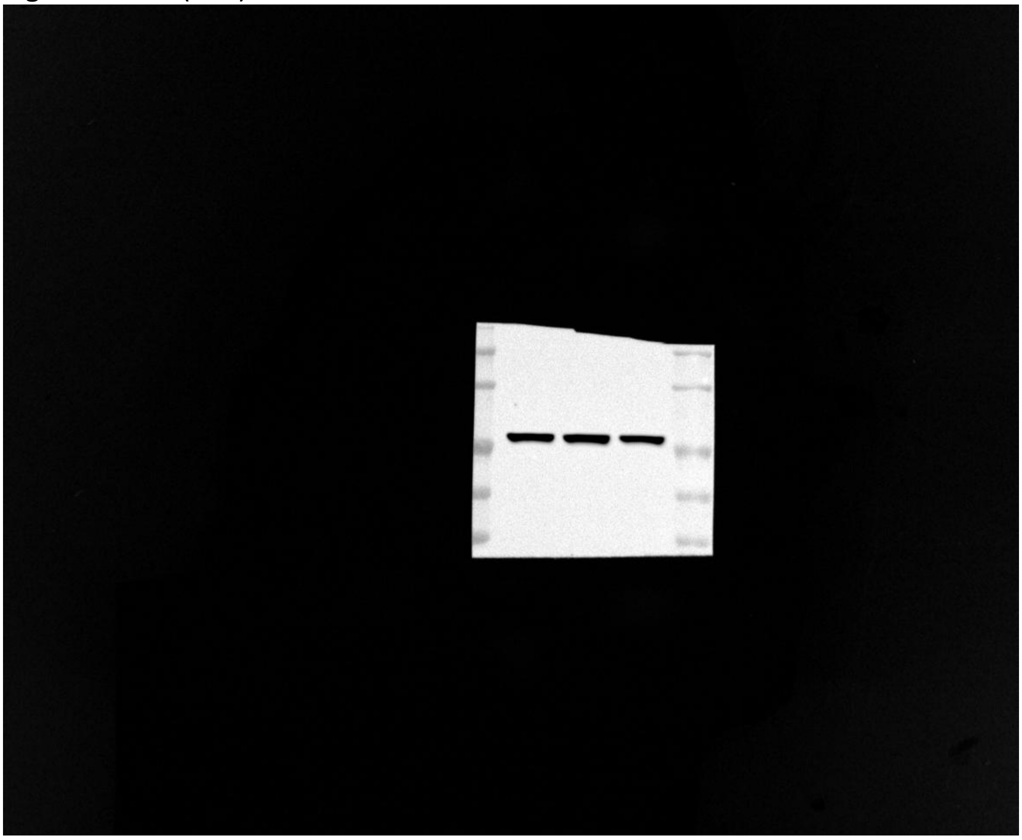

Figure4-e-WB(GAPDH)

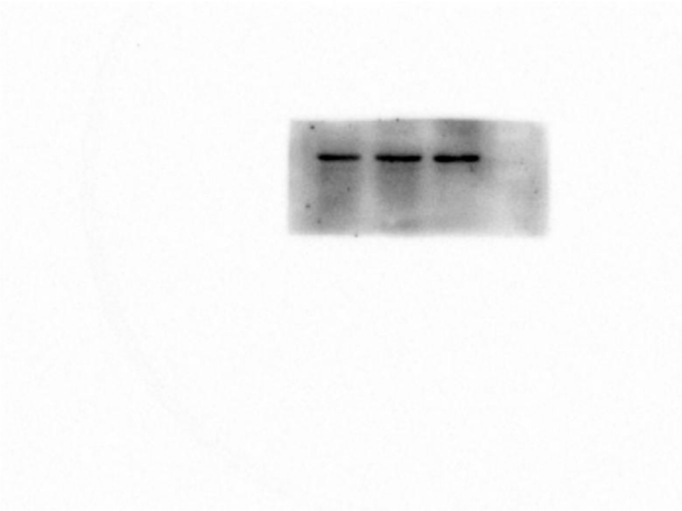

Figure4-e-WB(shTUG1→RRAGD)

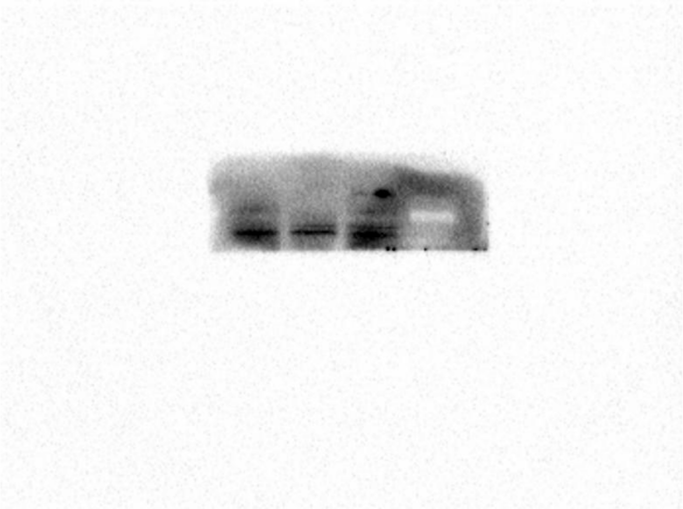

Figure6-e-WB(GAPDH)

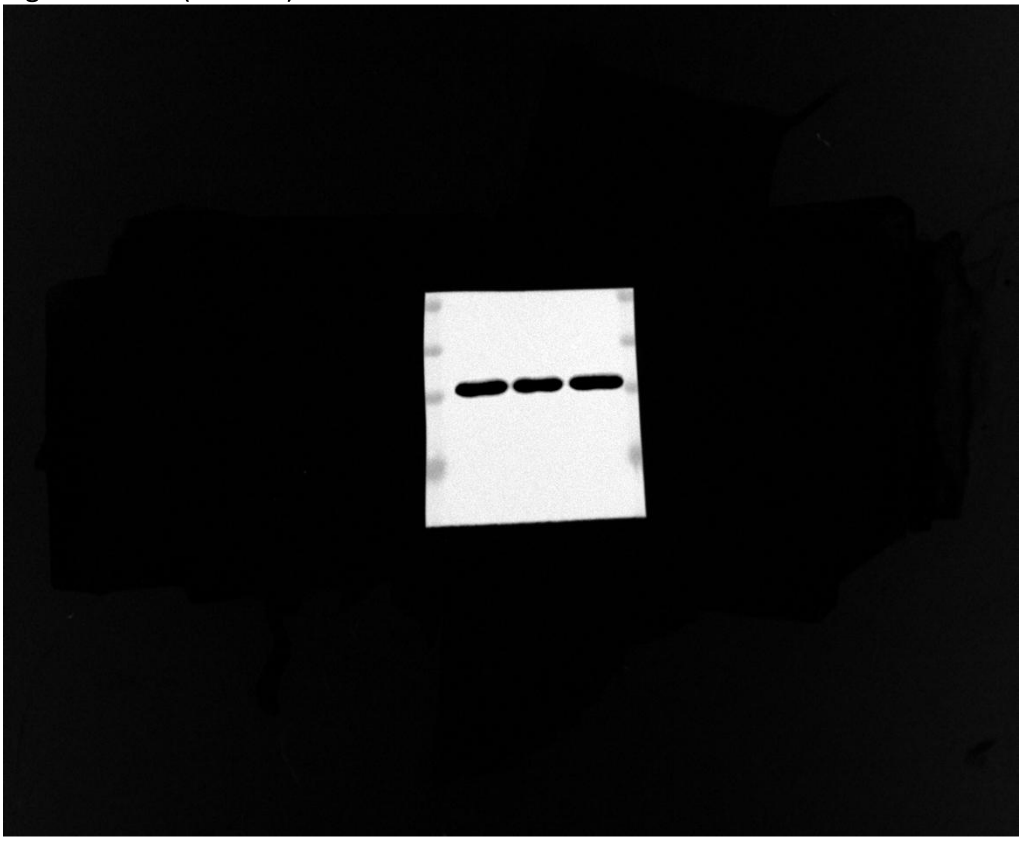

Figure6-e-WB(mTOR)

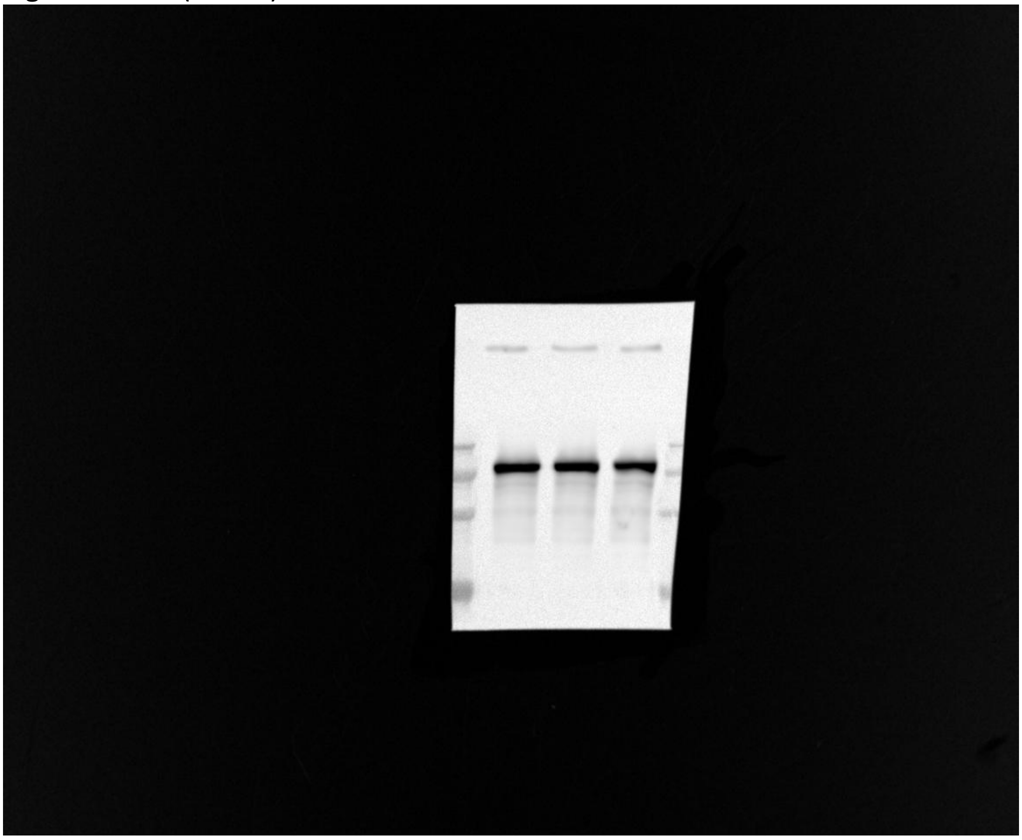

Figure6-e-WB(Figure70S6K)

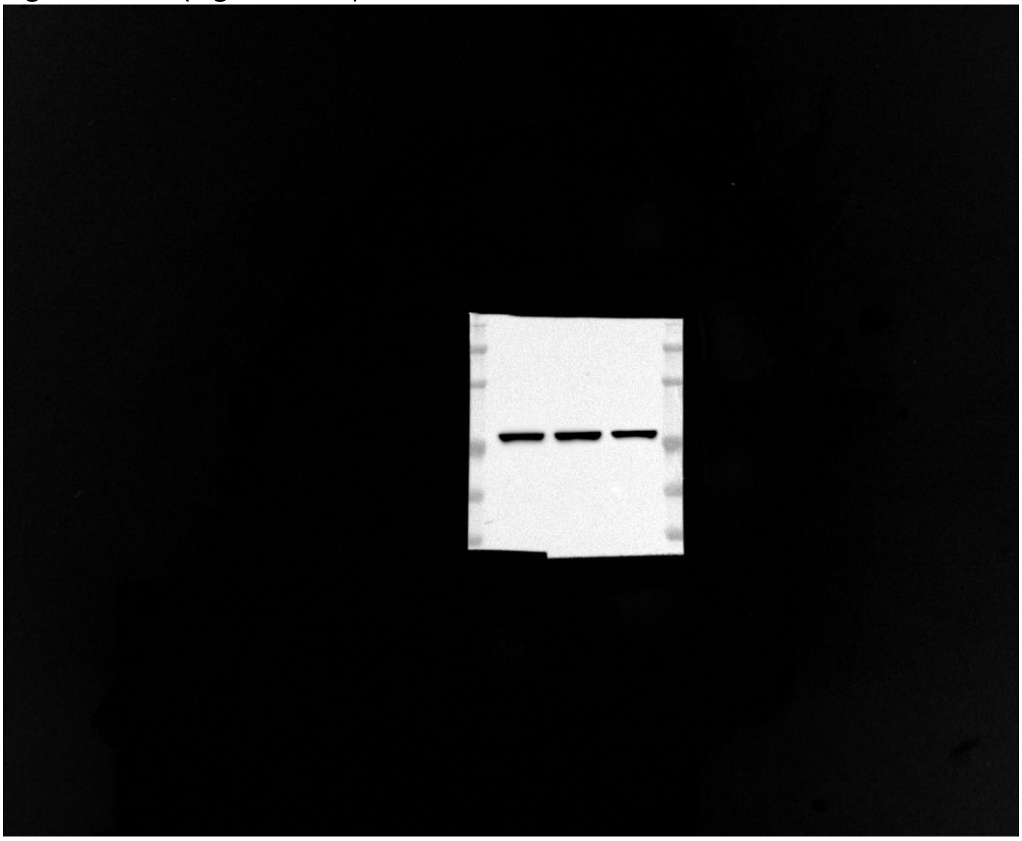

Figure6-e-WB(p-mTOR)

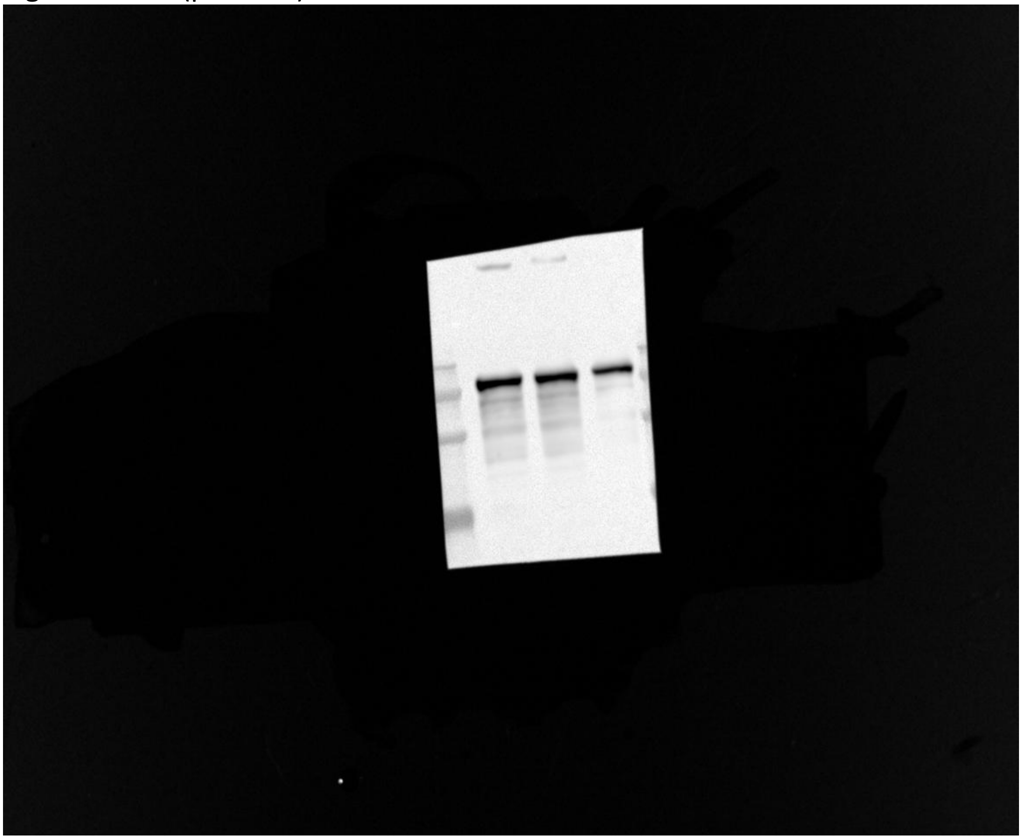

Figure6-e-WB(p-Figure70S6K)

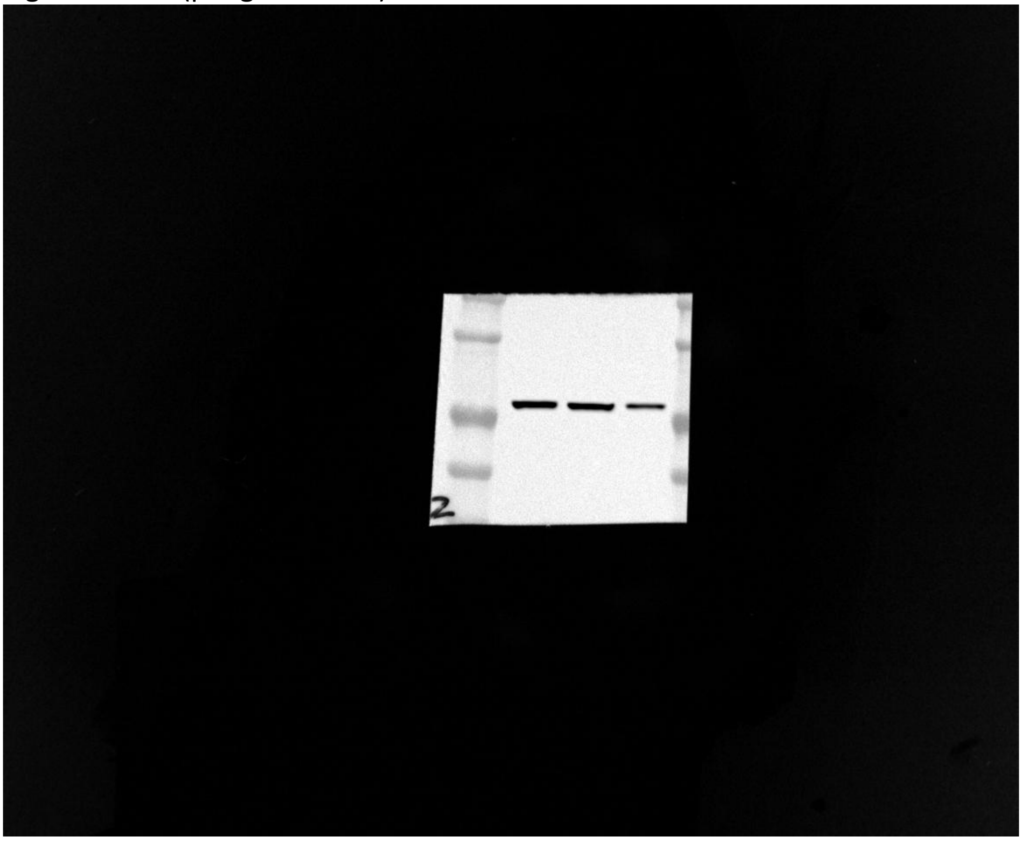

Figure6-f-WB(GAPDH)

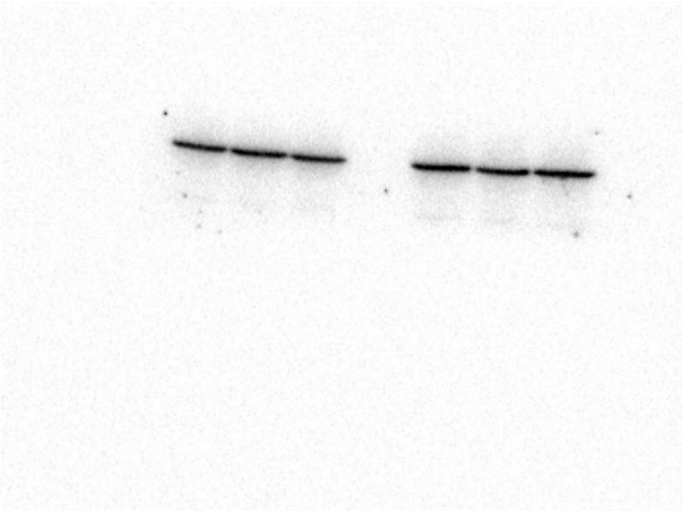

Figure6-f-WB(RRAGD)

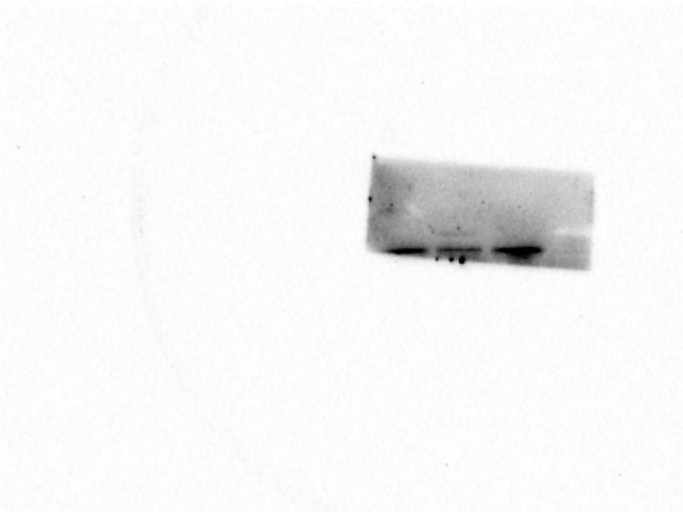

Figure6-j-WB(GAPDH)

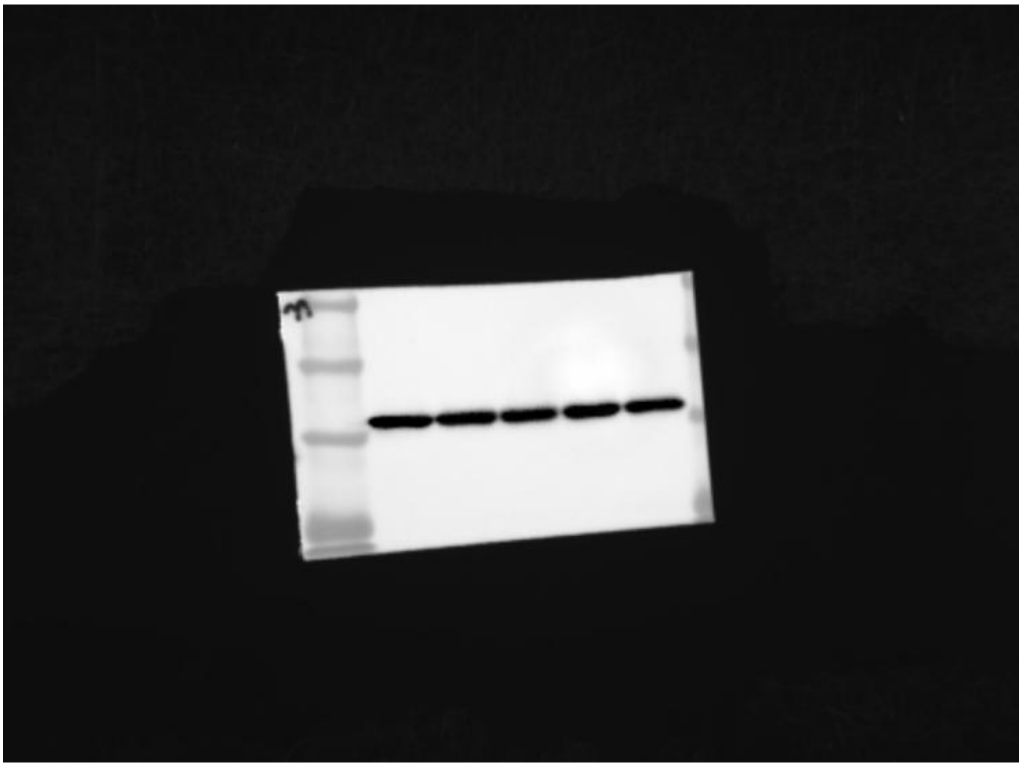

Figure6-j-WB(RRAGD)

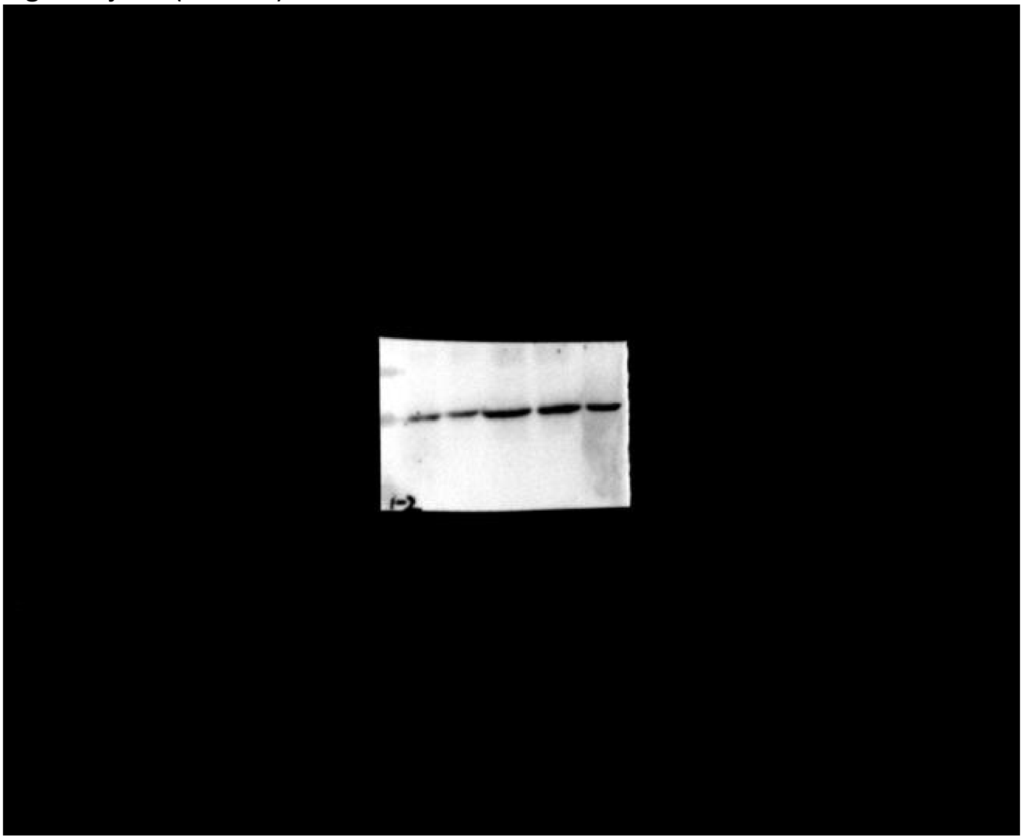

Figure7-e-WB(GAPDH)

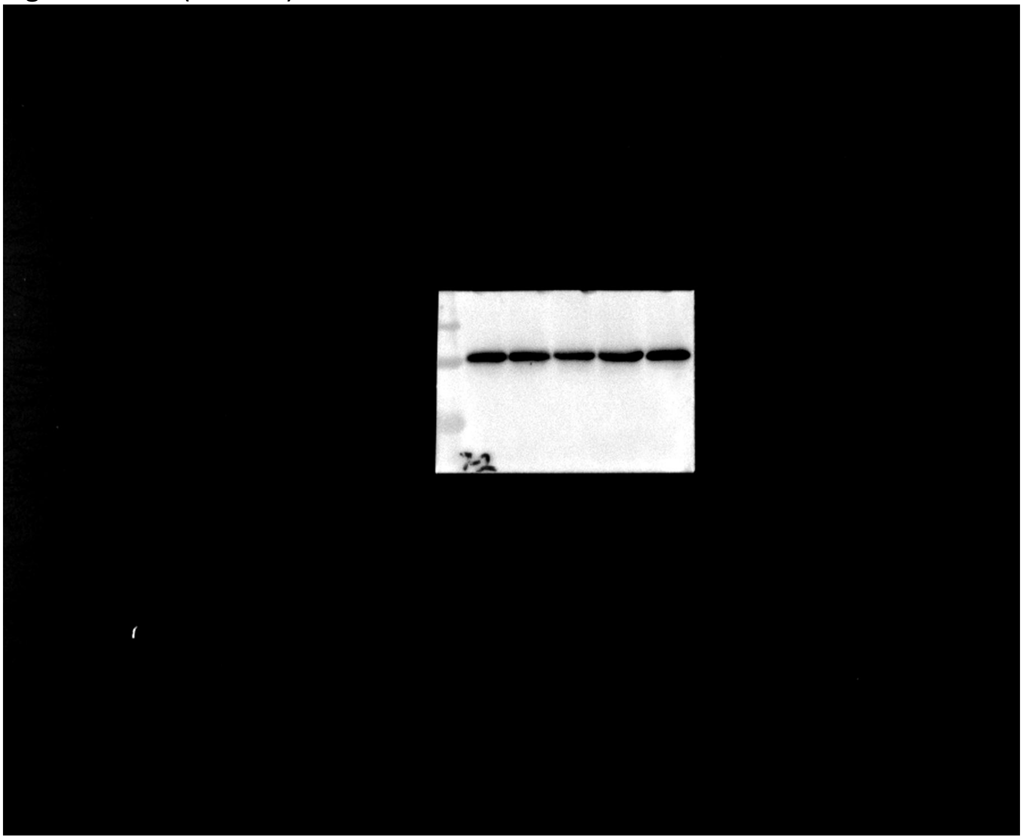

Figure7-e-WB(mTOR)

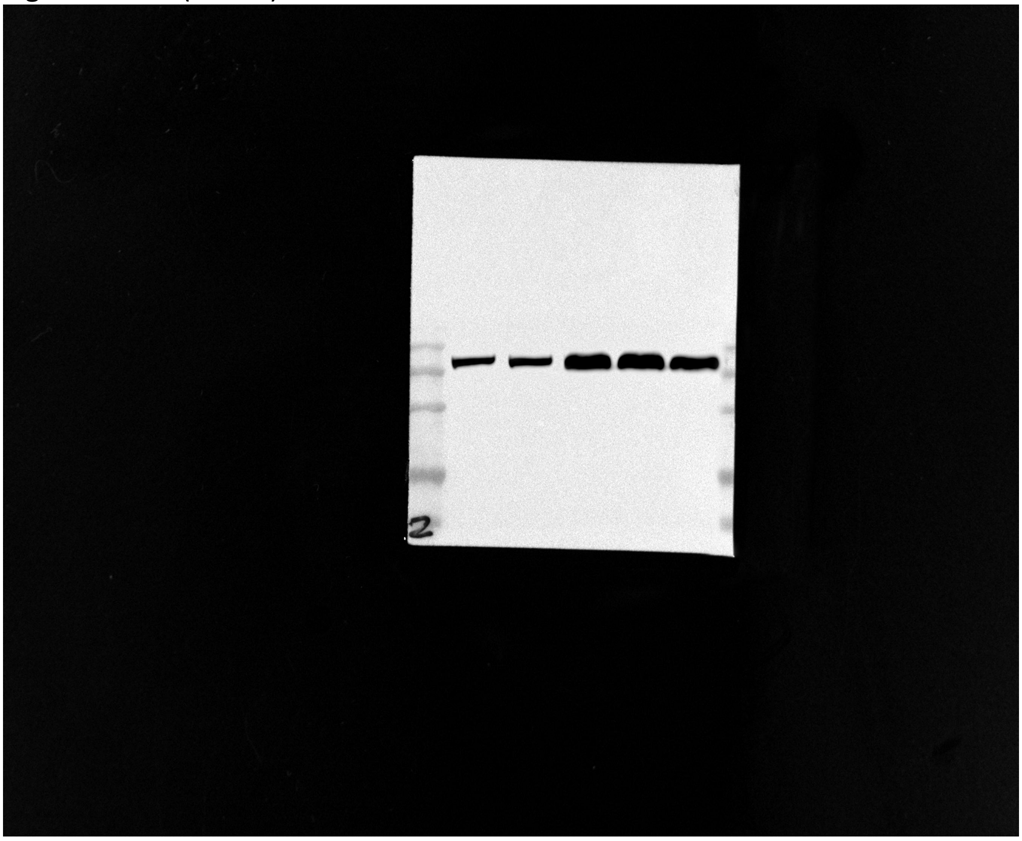

Figure7-e-WB(Figure70S6K)

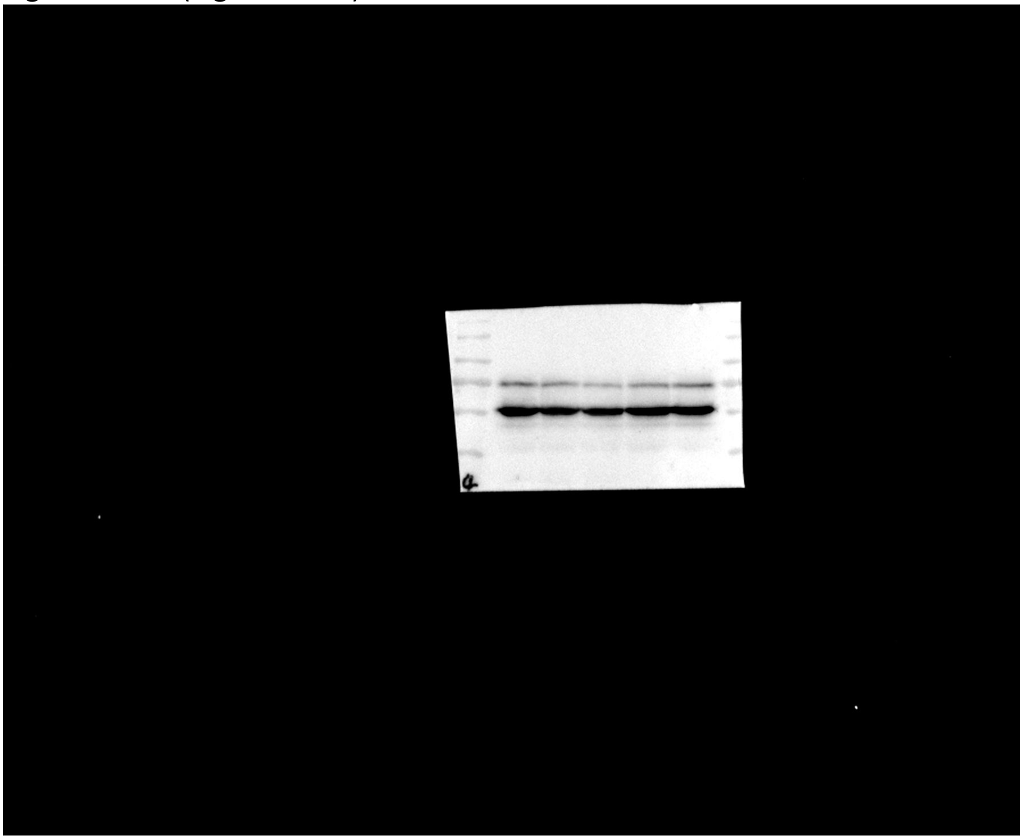

Figure7-e-WB(p-mTOR)

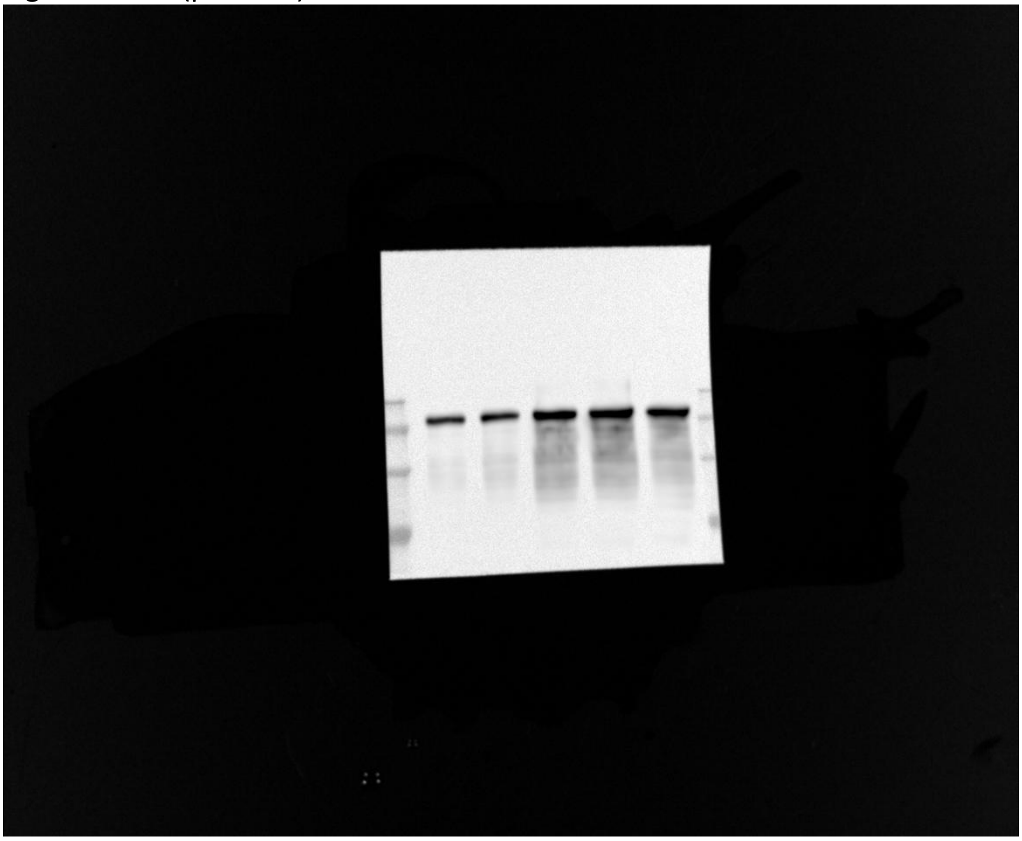

Figure7-e-WB(p-Figure70S6K)

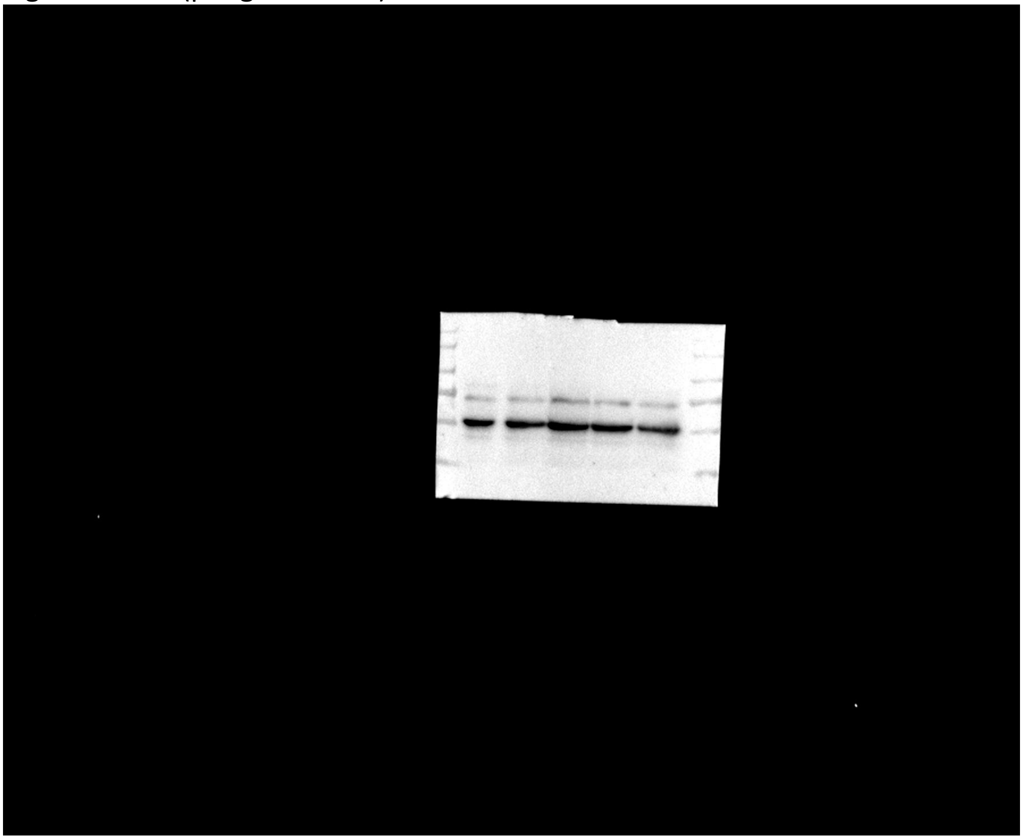

Figure8-e-WB(GAPDH)

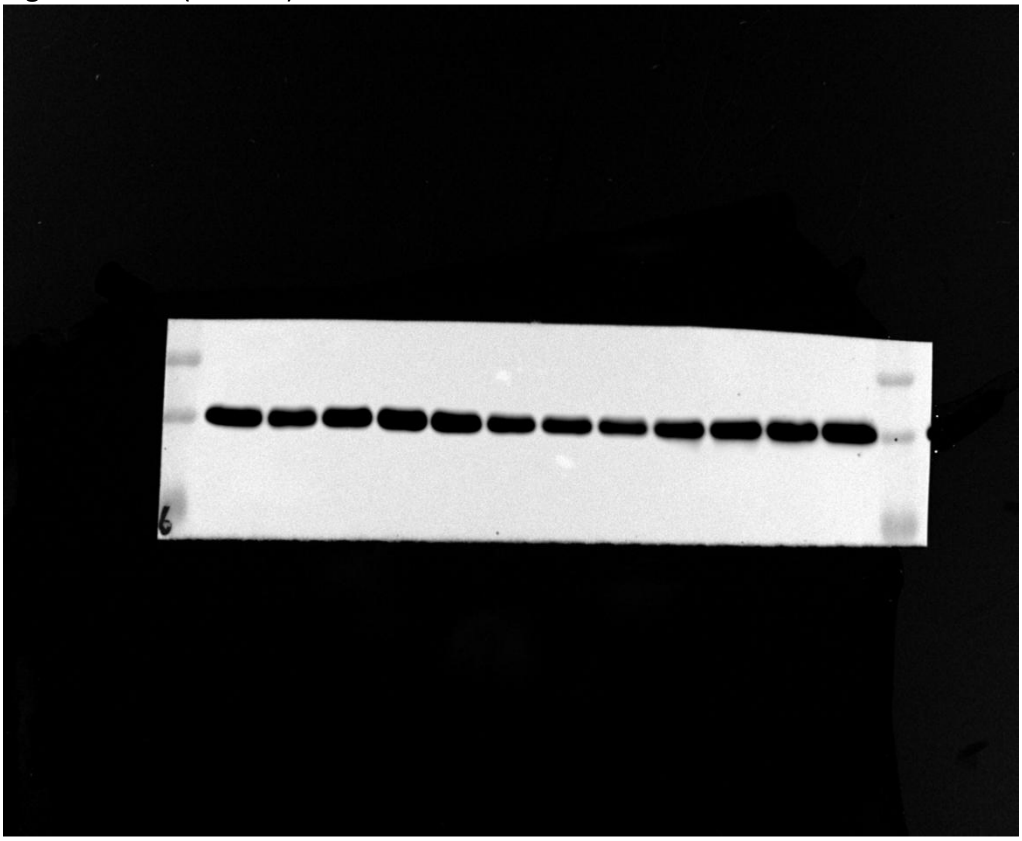

Figure8-e-WB(mTOR)

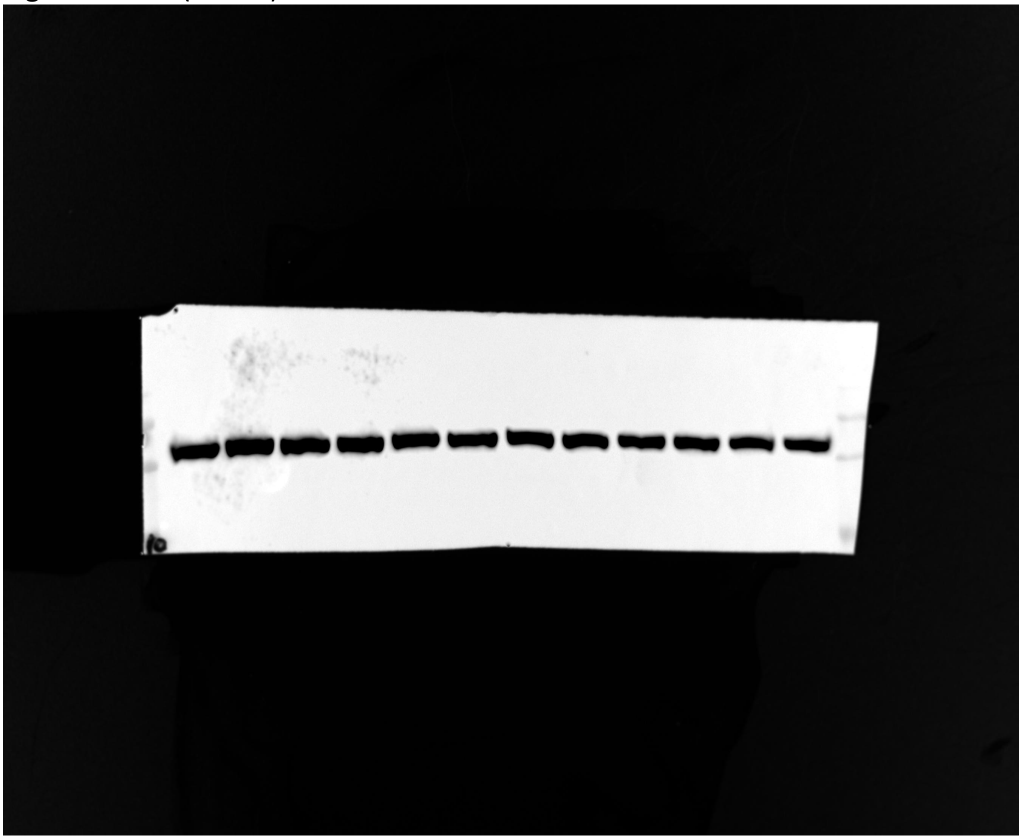

Figure8-e-WB(Figure70S6K)

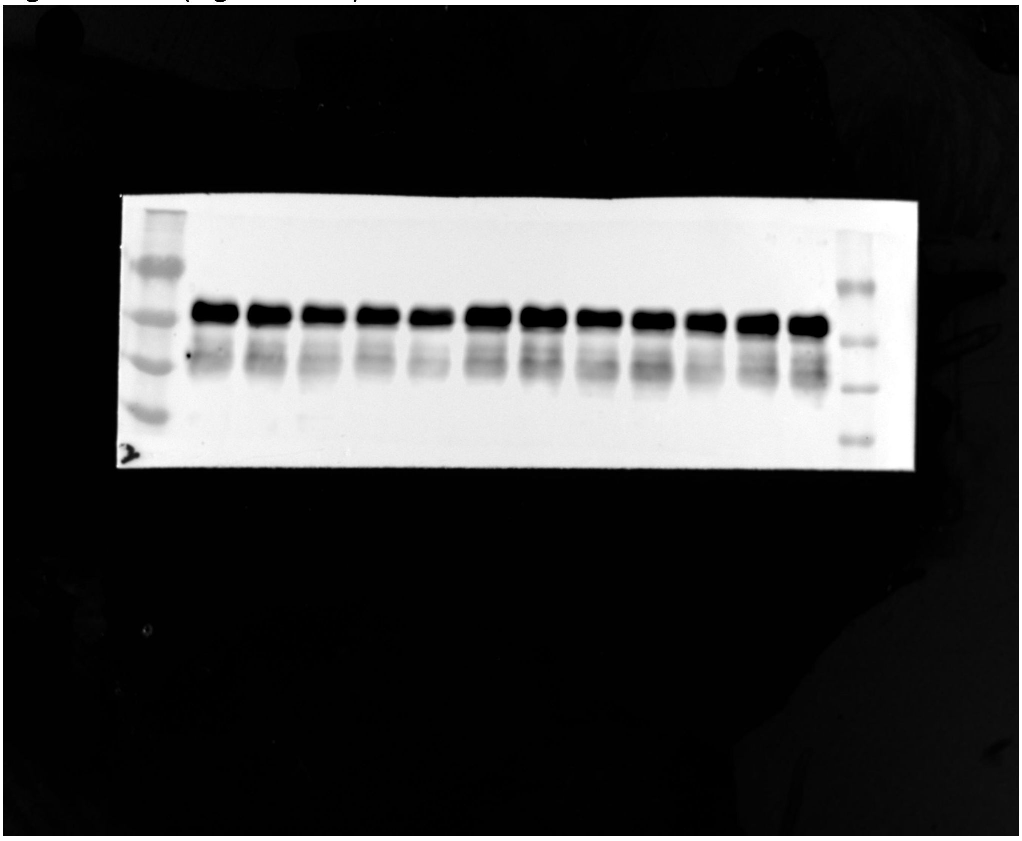

Figure8-e-WB(p-mTOR)

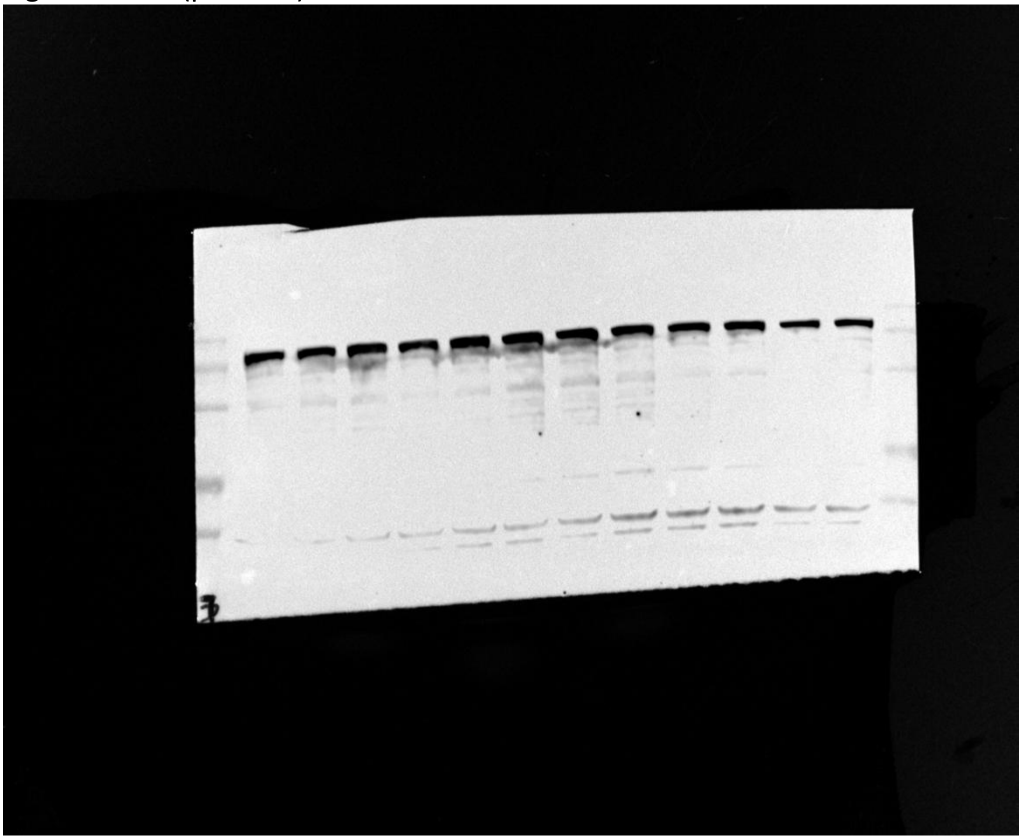

Figure8-e-WB(p-Figure70S6K)

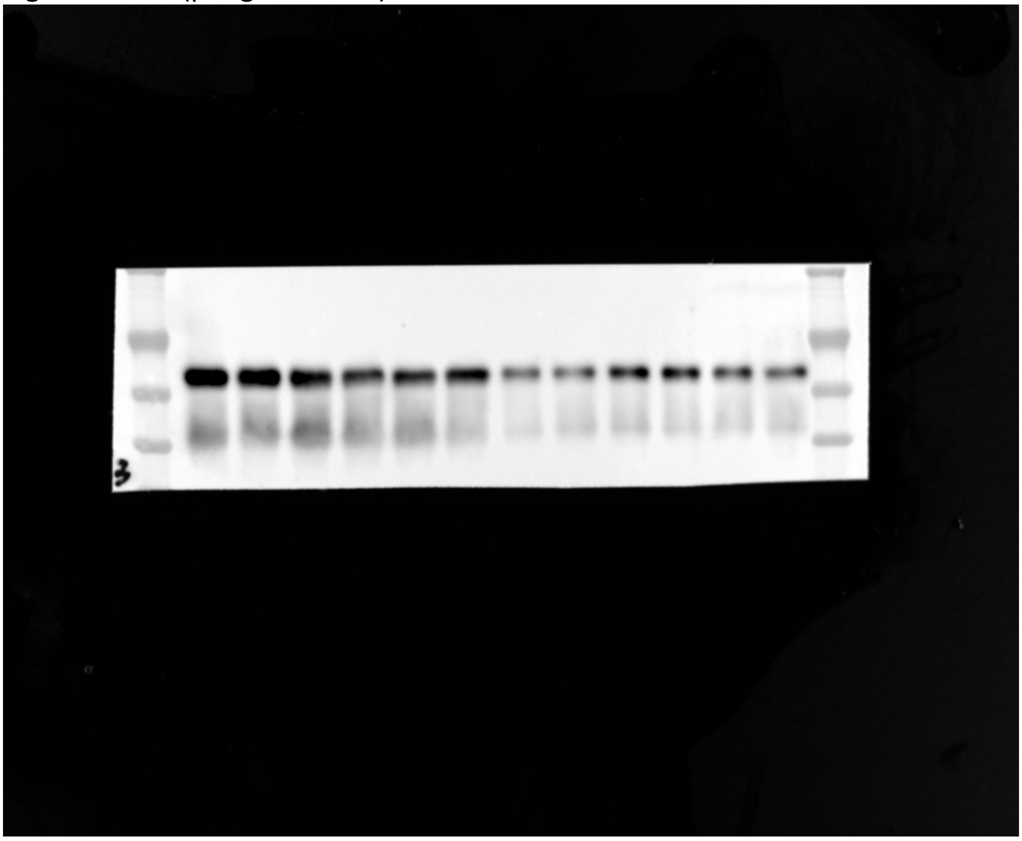

Figure8-e-WB(RRAGD)

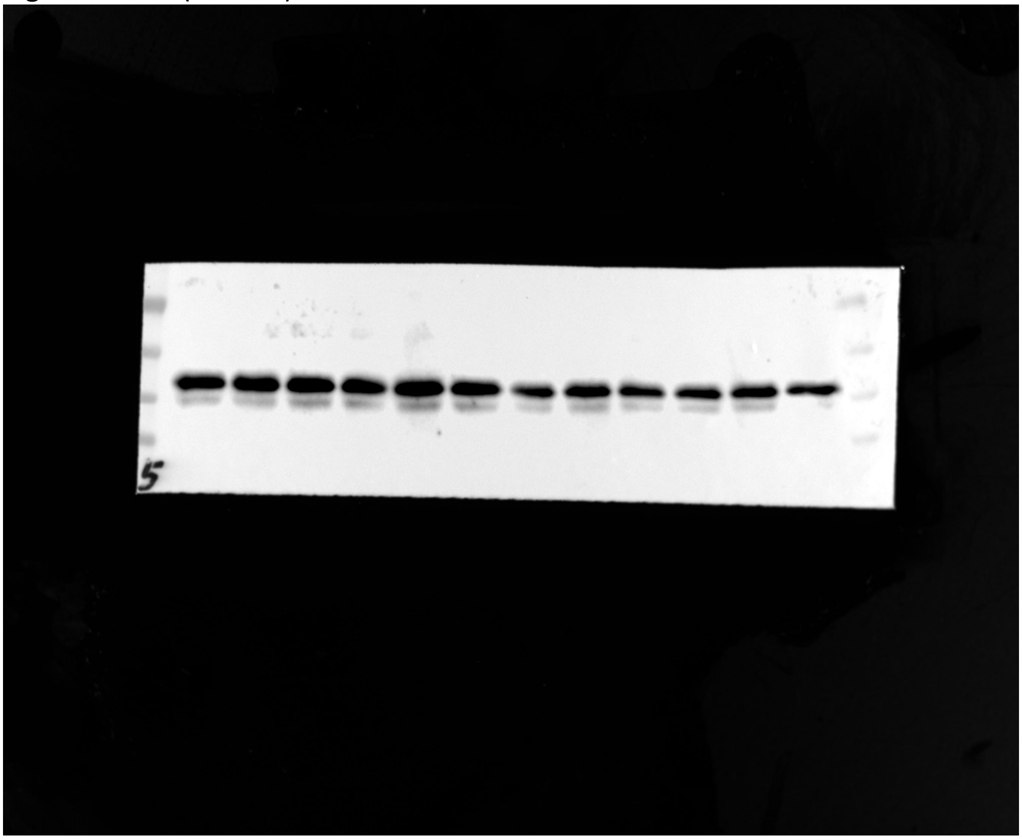

Supplement: Supplementary file 1 — Supplementary Information. [file 41598_2023_33976_MOESM1_ESM.pdf]
